# The pro-metastasis effect of circANKS1B in breast cancer

**DOI:** 10.1186/s12943-018-0914-x

**Published:** 2018-11-19

**Authors:** Kaixuan Zeng, Bangshun He, Burton B. Yang, Tao Xu, Xiaoxiang Chen, Mu Xu, Xiangxiang Liu, Huiling Sun, Yuqin Pan, Shukui Wang

**Affiliations:** 1 General Clinical Research Center, Nanjing First Hospital, Nanjing Medical University, Nanjing, 210006 Jiangsu China; 2 School of Medicine, Southeast University, Nanjing, 210096 China; 3 Sunnybrook Research Institute and Department of Laboratory Medicine and Pathology, Faculty of Medicine, University of Toronto, Toronto, M5S 1A1 Canada

**Keywords:** CircRNA, Breast cancer, Prognosis, Metastasis, EMT

## Abstract

**Background:**

Recent studies indicate that circular RNA (circRNA) plays a pivotal role in cancer progression. Here, we sought to investigate its role in breast cancer.

**Methods:**

CircANKS1B (a circRNA originated from exons 5 to 8 of the ANKS1B gene, hsa_circ_0007294) was identified by RNA-sequencing and validated by qRT-PCR and Sanger sequencing. Clinical breast cancer samples were used to evaluate the expression of circANKS1B and its associations with clinicopathological features and prognosis. Gain- and loss-of-function experiments in cell lines and mouse xenograft models were performed to support clinical findings and elucidate the function and underlying mechanisms of circANKS1B in breast cancer.

**Results:**

CircANKS1B was significantly up-regulated in triple-negative breast cancer (TNBC) compared with non-TNBC tissues and cell lines. Increased circANKS1B expression was closely associated with lymph node metastasis and advanced clinical stage and served as an independent risk factor for overall survival of breast cancer patients. Functional studies revealed that circANKS1B promoted breast cancer invasion and metastasis both in vitro and in vivo by inducing epithelial-to-mesenchymal transition (EMT), while had no effect on breast cancer growth. Mechanistically, circANKS1B abundantly sponged miR-148a-3p and miR-152-3p to increase the expression of transcription factor USF1, which could transcriptionally up-regulate TGF-β1 expression, resulting in activating TGF-β1/Smad signaling to promote EMT. Moreover, we found that circANKS1B biogenesis in breast cancer was promoted by splicing factor ESRP1, whose expression was also regulated by USF1.

**Conclusions:**

Our data uncover an essential role of the novel circular RNA circANKS1B in the metastasis of breast cancer, which demonstrate that therapeutic targeting of circANKS1B may better prevent breast cancer metastasis.

**Electronic supplementary material:**

The online version of this article (10.1186/s12943-018-0914-x) contains supplementary material, which is available to authorized users.

## Background

Breast cancer, the second common cancer in females, is a complex heterogeneous disease, which can be divided into four major molecular subtypes [1]. Of them, triple-negative breast cancer (TNBC), characterized by the loss of expression of estrogen receptors (ERs), progesterone receptors (PRs), and human epidermal growth factor receptor 2 (HER2), is more prone to metastasize to distant sites compared with other subtypes of breast cancer [2]. Metastasis is responsible for over 90% of the cases of mortality in patients with breast cancer, and current effective therapeutic agent targeting metastasis is still lacking owing to the spatiotemporal intratumor heterogeneity during metastasis [3]. Therefore, elucidation of the underlying mechanisms which contribute to metastasis is desperately needed to provide novel therapeutic strategies for patients with metastatic breast cancer.

Cancer metastasis is a complex and multi-step process [4]. Accumulating evidence shows that epithelial-to-mesenchymal transition (EMT) is the pivotal step for breast cancer cells to metastasis, whereby epithelial cells gradually lose polarity and adhesion capacity but gain mesenchymal traits with down-regulation of the epithelial biomarker E-cadherin and up-regulation of mesenchymal markers vimentin [5, 6]. Meanwhile, it is widely accepted that TGF-β signaling is a primary EMT inducer by activation of Smad complexes that translocate into the nucleus to regulate gene expression [7], which is critical for breast cancer progression and heterogeneity [8].

Recently, the roles of circular RNAs (circRNAs) in cancer have attracted much attention. CircRNAs, characterized by covalently closed loop structures without 5′-cup structure and 3′-polyadenylated tail, are highly conserved and stable [9, 10]. With the advent of high-throughput sequencing, circRNAs are now known to be not simply by-products of splicing errors but rather the product of a new type of regulated alternative splicing [11, 12]. CircRNAs are formed by back-splicing of exons or introns with gene-regulatory potency and both cis-elements (e.g., Alu elements) [13, 14] and trans-acting factors (e.g., Quaking and Muscleblind) [15, 16] participate in their biogenesis. Growing studies showed that circRNAs were involved in various cancer biological processes, including EMT and metastasis, via interaction with miRNA [17, 18]. For instance, circRNA-MYLK promoted bladder carcinoma metastasis by inducing EMT via sponging miR-29a [19]. CircSMARCA5 directly bound to miR-17-3p and miR-181b-5p to inhibit the metastasis of hepatocellular carcinoma [20]. And our previous study also demonstrated that circHIPK3 contributed to colorectal cancer metastasis through interacting with miR-7 [21]. Despite advancements in the study of circRNAs, the potential correlation between circRNAs and breast cancer metastasis is still unclear and remains to be further investigated.

In the present study, we characterized one novel circRNA originated from exons 5 to 8 of ANKS1B gene (hsa_circ_0007294, circANKS1B). Furthermore, the biogenesis, functions and mechanisms of circANKS1B in breast cancer were studied.

## Methods

### Patient population and clinical data

In total, 23 pairs of fresh frozen TNBC and adjacent normal tissues (cohort 1, 2), 165 formalin-fixed, paraffin-embedded (FFPE) breast cancer tissues and 40 normal tissues (cohort 3) were collected from Affiliated Nanjing First Hospital of Nanjing Medical University (Nanjing, China). Patients treated with any anti-tumour treatment before surgery were excluded. All histologic slides were independently identified by two pathologists. Of them, RNA-sequencing was performed in 3 pairs (cohort 1), 20 pairs (cohort 2) were used for circRNA validation and cohort 3 was used for quantification of circANKS1B in all breast cancer subtypes and normal tissues and analysis of the correlations between circANKS1B expression and breast cancer clinico-pathological parameters and outcome. The detail patient characteristics were described in Additional file 1: Table S1. Patient follow-up was performed in the outpatient department by phone or letter. Informed consent was obtained from each patient and the study was approved by the ethics committee of Nanjing First Hospital.

### RNA sequencing, identification and quantification of human circRNAs

The total RNA was extracted from three pairs of fresh frozen TNBC and adjacent normal tissues by using TRIzol reagent (Invitrogen, CA, USA), followed by treatment with the RiboMinus Eukaryote Kit (Qiagen, Valencia, CA) to delete ribosomal RNA according to the manufacturer’s guidelines. Next, the processed RNAs were subjected to perform deep sequencing with an Illumina HiSeq 3000 (Illumina, San Diego, CA).

The RNA-seq FASTQ reads were first aligned to the human reference genome (GRCh37/hg19) by TopHat2 [22]. The sequences that aligned contiguously and full length to the genomes were discarded. Then, the remaining reads were used to identify circRNAs [14]. SRPBM (spliced reads per billion mapping) was applied to normalize the counts of reads mapping across an identified backsplice, and differential expression analysis was conducted based on the previous method [23].

### Cell culture

Human normal breast epithelial cell line (MCF10A) and breast cancer cell lines (MCF-7, T47D, SK-BR-3, MDA-MB-231, MDA-MB-468 and BT549) were all purchased from American Type Culture Collection (Manassas, VA, USA). The culture method of MCF-10A cell was described in the previous study [24]. Other cell lines were cultured in DMEM or RPMI1640 media plus 10% fetal bovine serum (Gibco, Vienna, Austria) at 37 °C with 5% CO2. All cell lines were authenticated and tested for mycoplasma every 4 months using MycoAlert Mycoplasma Detection Kit (Lonza, Switzerland).

### Antibodies and reagents

The antibodies we used are as follows: anti-E-cadherin (Abcam # ab40772), anti-Vimentin (Abcam # ab92547), anti-Fibronectin (Proteintech # 15613–1-AP), anti-AGO2 (Abcam # ab57113), anti-USF1 (Santa Cruz # sc-390,027), anti-RNA polymerase II (Santa Cruz # sc-47,701), anti-TGF-β1 (Abcam # ab92486), anti-ESRP1 (Abcam # ab107278), anti-p-Smad2 (Cell Signaling Technology # 8828), anti-p-Smad3 (Cell Signaling Technology # 9520), anti-Smad2/3 (Cell Signaling Technology # 8685), anti-GAPDH (Proteintech # 10494–1-AP) and anti-β-actin (Cell Signaling Technology # 4970). Actinomycin D and RNase R were purchased from Sigma-Aldrich (St Louis, MO, USA) and Epicentre Technologies (Madison, WI, USA), respectively. The TGF-β receptor type I/II inhibitor LY2109761 was obtained from Selleckchem Chemicals (Houston, TX, USA).

### RNA extraction and qRT-PCR

Total RNA was isolated from tissue samples and cultured cells with TRIzol reagent (Invitrogen). RNA quantity was tested by a SmartSpec Plus spectrophotometer (Bio-Rad). The Hairpin-itTM MicroRNAs Quantitation PCR Kit (Gene-Pharma, Shanghai, China) was used to measure the expression of miRNA, and U6 was used as the internal control. To detect circRNA and mRNA, 1 μg of RNA was reverse transcribed to cDNA using the PrimeScript RT Reagent (Takara, Otsu, Japan) and then subjected to qPCR using the SYBR Premix Ex Taq™ (Takara). GAPDH was used as an internal control. The 2^−ΔΔCt^ method was applied to quantify gene expression. The primer sequences were shown in Additional file 1: Table S3.

### Oligonucleotide transfection

siRNA, miRNA mimics and inhibitors were purified and synthesized by RiboBio (Guangzhou, China) or Gene-Pharma (Shanghai, China). Transfection was performed using Lipofectamine 2000 reagent (Invitrogen). The RNA sequences used are listed in Additional file 1: Table S3.

### Plasmids construction and stable transfection

The circRNA-expressing vectors were constructed as described previously [15, 25]. In brief, full-length of human circANKS1B along with 1.2 kb endogenous 5′-flanking intron and 0.8 kb 3′ -flanking intron was subcloned into the pCDH-CMV-GFP vector (Geenseed Biotech, Guangzhou, China). And the sequence of the 5′-flanking intron was copied and inversely inserted the downstream of 3′-flanking intron. Besides, the canonical splicing site (AG-GT) was reserved for correct splicing. For circANKS1B minigene reporters, the inversely inserted 5′-flanking intron was deleted. For SYT8 and Snail minigenes, genomic regions comprising three exons and two introns with or without ESRP1 binding site (GGT-rich) were synthesized and inserted into the pCDH-CMV-GFP vectors. For USF1, TGF-β1 and ESRP1-expressing vectors, the full-length ORF sequences of these three genes were respectively subcloned into the pLenti-CMV-GFP vectors (ABM, Richmond, BC, Canada). And two si-circANKS1B sequences were subcloned into pGLV3/H1/GFP/Puro vectors to construct sh-circANKS1B for animal studies. All constructs were verified by sequencing. Lentiviral particles carrying above-mentioned vectors were generated in HEK293T cells. Then, breast cancer cells were infected with lentivirus at a multiplicity of infection (MOI) of 30, followed by selection with 1–2 μg/mL puromycin.

### Fluorescence in situ hybridization

The FISH assay was carried out using Fluorescent In Situ Hybridization Kit (Gene-Pharma, Shanghai, China) based on the manufacturer’s protocols. The hybridization was performed with Cy3-labeled circANKS1B and FAM-labeled miR-148a-3p or miR-152-3p probes (Gene-Pharma), followed by analysis using a confocal microscopy. The probe sequences were shown in Additional file 1: Table S3.

### Wound healing and transwell assays

For wound healing assay, breast cancer cells were seeded into a 6-well plate and scraped using a sterile pipette tip. Images were obtained using an inverted microscope at 0 and 24 h, and then analyzed by Image J. For transwell migration and invasion assays, breast cancer cells were seeded into the upper chamber without (migration assay) or with (invasion assay) the matrigel (Corning, NY, USA). After 24 h of incubation, non-migrated or invaded cells were scraped off with a cotton swab and cells on the bottom of the chamber were fixed, stained, and counted.

### Immunoblot analysis

Breast cancer cells were washed and then lysed in RIPA lysis buffer. After that, protein extracts were boiled for 5 min, separated on a 10% SDS-PAGE and transferred to a PVDF membrane (Millipore, Schwalbach, Germany). Subsequently, the membrane was incubated with corresponding primary antibody at 4 °C overnight. Next, the membrane was washed five times and incubated with secondary antibody, and bands were then visualized.

### RNA immunoprecipitation

RNA immunoprecipitation (RIP) assay was performed using Magna RIP™ RNA-binding protein immunoprecipitation kit (Millipore) according to the manufacturer’s guidelines with minor modifications. Briefly, the magnetic beads were incubated with 5 μg anti-AGO2 or anti-ESRP1 antibodies for 30 min at room temperature to generate antibody-coated beads. Breast cancer cells (2 × 10^7^) were lysed in 100 μl RIP lysis buffer and then diluted with 900 μl RIP immunoprecipitation buffer and incubated with the antibody-coated beads overnight at 4 °C. After that, beads were washed six times using RIP wash buffer. The immunoprecipitates were treated with Proteinase K at 55 °C for 30 min. And the isolated RNA was extracted using TRIzol regent (Invitrogen), followed by qRT-PCR.

### Biotinylated RNA pull-down assay

The RNA pull-down assay was conducted as described previously [21, 26]. Briefly, for the assay of pulling down miRNA by circRNA, breast cancer cells (1 × 10^7^) were lysed and incubated with biotinylated**-**circANKS1B probe that was pre-bound on C-1 magnetic beads (#65001, Life Technologies) at 4 °C overnight. Next, beads were eluted with rotation at 37 °C for 30 min. The bound RNAs were extracted for qRT-PCR. For the assay of pulling down circRNA by miRNA, breast cancer cells with circANKS1B overexpression were respectively transfected with biotinylated wild-type or mutant miR-148a-3p/ miR-152-3p mimics. 48 h later, the cells were collected and incubated with C-1 magnetic beads on the rotator at 4 °C for 3.5 h. And then washed five times and the bound RNAs were extracted for qRT-PCR. The probe sequences were described in Additional file 1: Table S3.

### Luciferase reporter assay

The circANKS1B or USF1 3′ UTR sequences containing wild-type or mutant miR-148a/152-3p binding sites were synthesized and respectively inserted into pmirGLO luciferase reporters (Promega) between Sacl and Sall restriction sites, after which cotransfected with miR-148a/152-3p mimics or control mimics into breast cancer cells using Lipofectamine 2000. For the promoter of TGF-β1 luciferase reporter assay, the wild-type or mutant full-length TGF-β1 promoter construct and six truncation constructs were respectively inserted into pGL3-basic vectors (Promega) between Sacl and Xhol restriction sites, and then cotransfected with USF1 overexpression vector and pRL-TK into breast cancer cells by Lipofectamine 2000. After 48 h, the luciferase activities were tested by the dual-luciferase reporter assay kit (Promega).

### Chromatin immunoprecipitation assay

The Chromatin immunoprecipitation (ChIP) assay was carried out as described previously [21]. In brief, breast cancer cells were collected and sonicated to generate DNA fragments of 200–1000 bp and then incubated with anti-USF1, anti-RNA polymerase II (positive control) or anti-IgG antibody (negative control) overnight at 4 °C. Immunoprecipitated DNA was extracted and subjected to PCR analysis. The primer sequences were listed in Additional file 1: Table S3.

### Immunohistochemistry

Immunohistochemistry (IHC) was performed as described previously [27] with anti-USF1 and anti-ESRP1 antibody in the formalin-fixed, paraffin-embedded breast cancer tissue sections (*n* = 165).

### Animal studies

MCF-7 cells with circANKS1B overexpression, MDA-MB-231 cells with circANKS1B knockdown and their respective control vectors were respectively tail-vein (2 × 10^6^ cells) injected into the female BALB/c nude mice (8 mice in each group). Six weeks later, mice were euthanized. The lungs were collected and metastatic nodules were counted after H&E staining. And the animal studies were approved by the Animal Care Committee of Nanjing Medical College (acceptance no.: SYXK20160006).

### Analysis of public databases

The raw gene expression data in breast cancer (*n* = 1109) were downloaded from The Cancer Genome Atlas (TCGA) database (https://cancergenome.nih.gov/). Then, the expression values (counts) of USF1, TGF-β1 and ESRP1 were obtained by using R software. To evaluate the prognostic value of USF1 and ESRP1 in breast cancer patients, we analyzed the Kaplan-Meier plotter database (http://kmplot.com/analysis/). The median expression values of USF1 and ESRP1 were set to the cutoff values of the overall and distant metastasis-free survival curves.

### Statistical analysis

The differences between groups were determined by Student’s t-test or one-way ANOVA. Kaplan-Meier plot and Cox proportional hazards model were respectively applied to determine the patient survival and independent factors. And the correlations were measured by Spearman correlation coefficients. A two-sided *p* < 0.05 was considered statistically significant.

## Results

### CircANKS1B is highly expressed in TNBC and predicts poor prognosis

We characterized circRNA transcripts by performing RNA-seq on ribosomal RNA-depleted total RNA from three pairs of TNBC and adjacent normal tissues. In all, 69,815 distinct circRNAs were found in this study and 87% were derived from exons, and the others were derived from introns, intergenic region and 3′ or 5′ UTR, etc. (Additional file 1: Figure S1A). The length of most circRNAs was less than 1300 nucleotides (nt) and the median length was 508 nt (Additional file 1: Figure S1B). Furthermore, after excluding the circRNAs with very low abundance (average RPM < 0.1) and less back-spliced reads (< 2 back-spliced reads), we identified 5033 differentially expressed circRNAs (fold change ≥2 and *p* < 0.05). Among them, 3726 circRNAs were significantly down-regulated and 1307 circRNAs were significantly up-regulated in TNBC tissues compared with adjacent normal tissues. Besides, we found that there were 4227 overlapped circRNAs and 806 novel circRNAs compared with circBase [28] (92,061 human circRNAs).

**Fig. 1:**
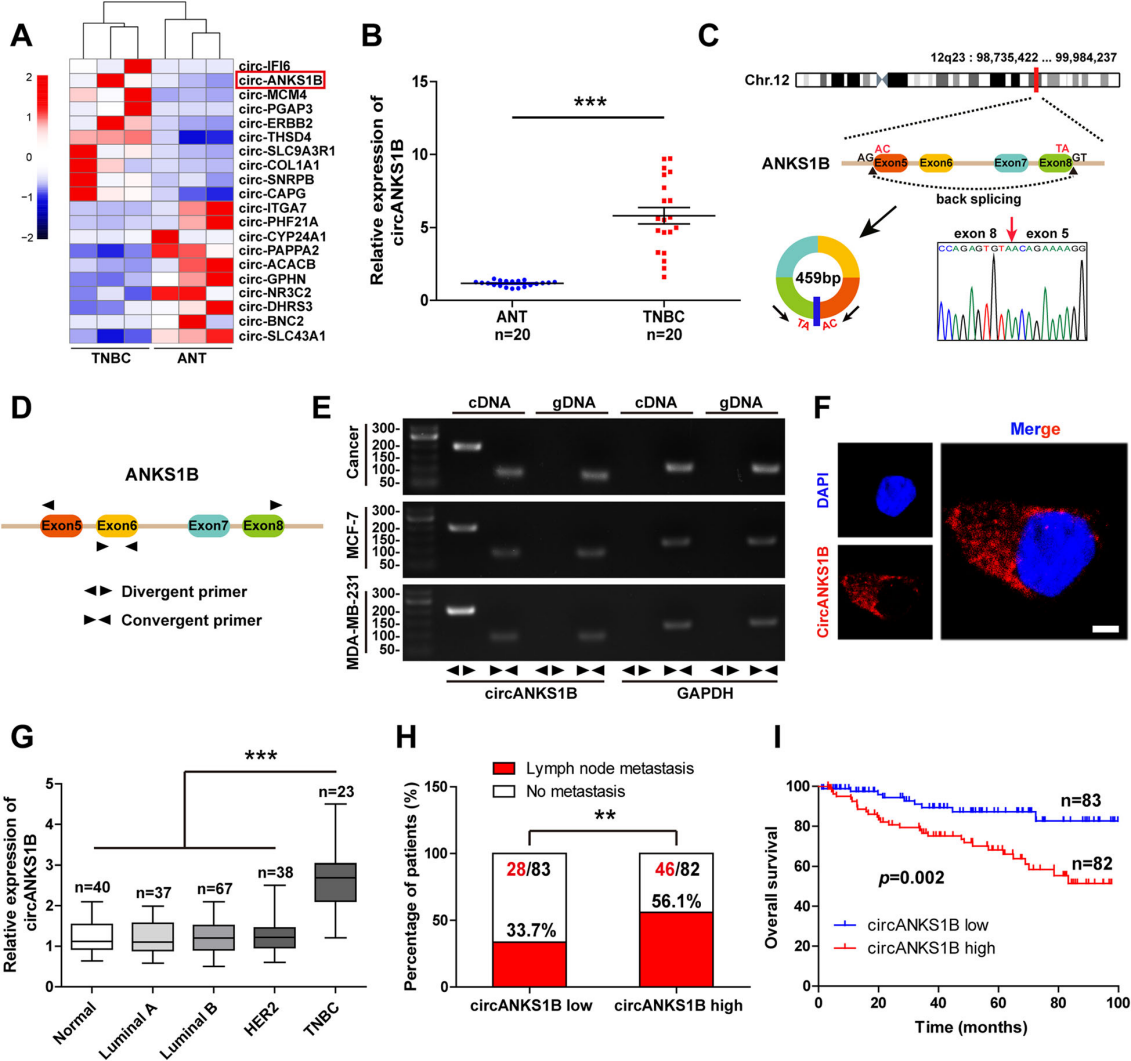
Identification of circular RNAs by RNA-seq and the characteristics of circANKS1B in breast cancer. **a** Heat map and hierarchical clustering analysis of the top ten up-regulated and down-regulated circRNAs in TNBC compared with adjacent normal tissues. Red colour represents high expression level, and blue colour represents low expression level. **b** qRT-PCR analysis of the expression of circANKS1B in twenty pairs of TNBC and adjacent normal tissues. **c** The genomic loci of ANKS1B gene and circANKS1B. Red arrow indicates the back-splicing of ANKS1B exon 5 to exon 8 confirmed by Sanger sequencing. The spliced mature sequence length of circANKS1B is 459 bp. **d** The schematic illustrates the targeted regions for qRT-PCR primers (Divergent primers for detecting circANKS1B and convergent primers for detecting linear ANKS1B). **e** RT-PCR for the analysis of the existence of circANKS1B in breast cancer tissue and cells. Divergent primers detected circANKS1B in cDNA but not genomic DNA (gDNA). GAPDH was used as the negative control. **f** Fluorescence in situ hybridization (FISH) assay was carried out to determine the subcellular localization of circANKS1B. circANKS1B probe was labeled with Cy3 (red), nuclei were stained with DAPI (blue). Scale bar = 10 μm. **g** qRT-PCR for the expression of circANKS1B in 40 normal tissues and 165 breast cancer tissues. **h** Analysis of circANKS1B expression in breast cancer patients with or without lymph node metastasis. Using median circANKS1B value as cutoff. **i** Kaplan-Meier survival curves of breast cancer patients with low and high circANKS1B expression. Using median circANKS1B value as cutoff. **p < 0.01, ***p < 0.001

Next, we narrowed the scope of the analysis to the 20 most aberrantly changed circRNAs (ten mostly increased and decreased circRNAs) (Fig. 1a). The additional 20 pairs of TNBC and adjacent normal tissues were used to validate the expression of these circRNAs. The qRT-PCR analysis showed that 5 circRNAs were identified to be significantly dysregulated, including 3 up-regulated circRNAs (circPGAP3, circANKS1B and circTHSD4) and 2 down-regulated circRNAs (circCYP24A1 and circACACB) (Fig. 1b and Additional file 1: Figure S2A-D). We then focused on studying circANKS1B due to its greatest change (approximately 6-fold) between TNBC and adjacent normal tissues (Fig. 1b). circANKS1B (Hsa_circ_0007294) arose from exons 5 to 8 of the ANKS1B gene, we thus termed it circANKS1B and its spliced mature sequence length is 459 bp (Fig. 1c). The convergent and divergent primers were designed to amplify ANKS1B mRNA and circANKS1B, respectively (Fig. 1d). RT-PCR results showed that circANKS1B was only amplified by divergent primers in cDNA, but not in gDNA (Fig. 1e). Next, we assessed the stability and localization of circANKS1B. After treatment with Actinomycin D or RNase R, we found circANKS1B was more stable than linear ANKS1B (Additional file 1: Figure S2E-G). And FISH analysis demonstrated that circANKS1B predominately localized in the cytoplasm (Fig. 1f).

To better understand the role of circANKS1B in breast cancer, the additional 165 breast cancer tissues (including all subtypes of breast cancer) and 40 normal tissues were collected to measure circANKS1B expression by qRT-PCR. Of note, circANKS1B was markedly up-regulated in TNBC compared with other subtypes of breast cancer and normal tissues (Fig. 1g). Increased circANKS1B expression was significantly associated with lymph node metastasis (*p* = 0.004) (Fig. 1h) and advanced clinical stage (*p* = 0.013) (Additional file 1: Table S1). Furthermore, Kaplan-Meier plots showed that high circANKS1B expression was closely associated with poor outcome (log-rank test, *p* = 0.002) (Fig. 1i). Lastly, the Cox proportional hazard model was applied to determine the prognostic value of circANKS1B, and results showed that circANKS1B high expression was an independent predictor of worse overall survival in patients with breast cancer (hazard ratio = 3.29, *p* = 0.008) (Additional file 1: Table S2). These data indicate that circANKS1B dysregulation may contribute to breast cancer development and progression.

### CircANKS1B, but not linear ANKS1B, promotes cell migration, invasion, and metastasis in breast cancer by inducing EMT

**Fig. 2:**
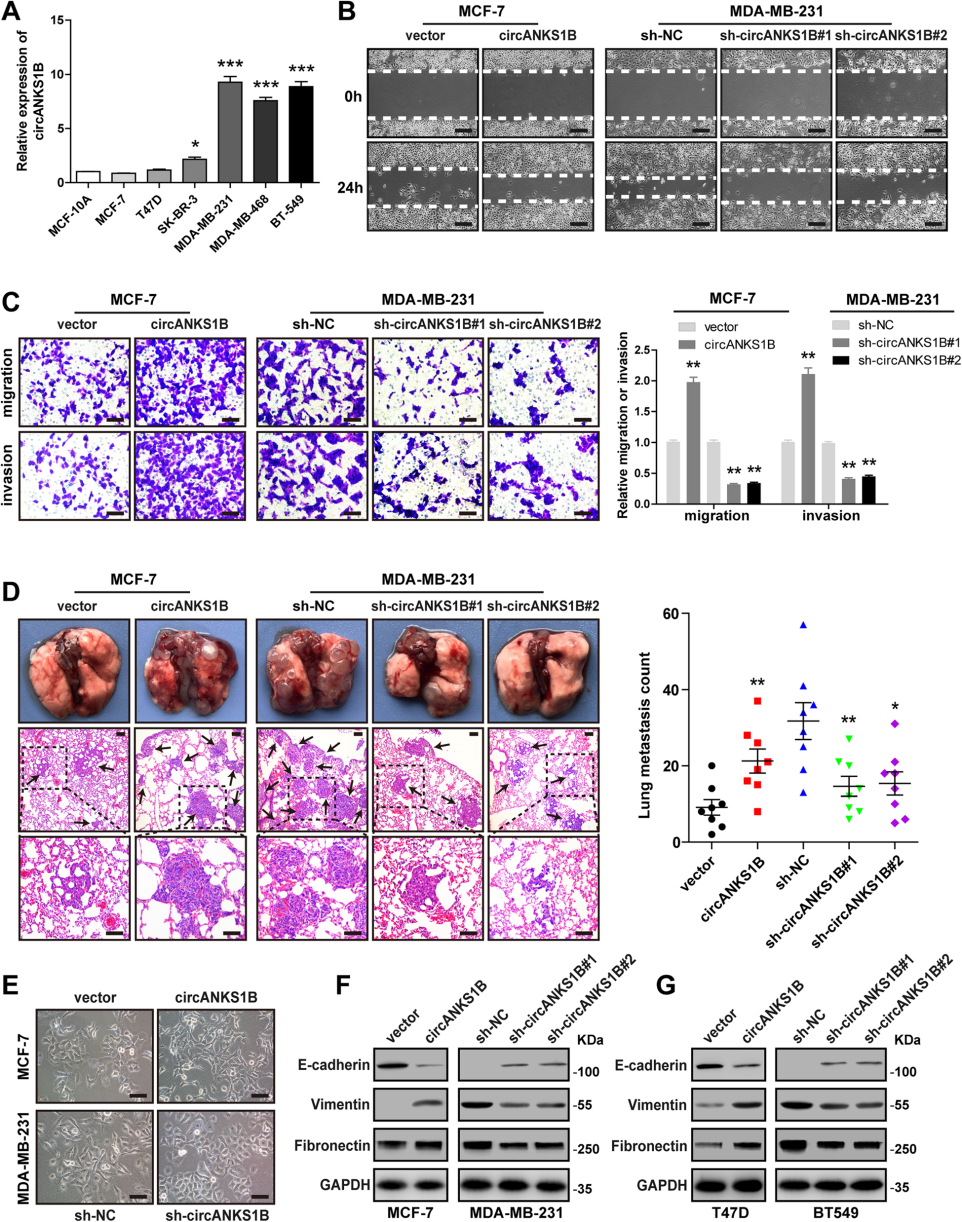
CircANKS1B enhances the migratory, invasive and metastatic capabilities of breast caner cells by inducing EMT. **a** qRT-PCR for the expression of circANKS1B in breast epithelial cell and breast cancer cells. **b** Wound healing assays for MCF-7 cells with circANKS1B overexpression and MDA-MB-231 cells with circANKS1B knockdown. Representative images are shown at 0 and 24 h after wound creation. Scale bar = 20 μm. **c** Transwell migration and matrigel invasion assays for MCF-7 cells with circANKS1B overexpression and MDA-MB-231 cells with circANKS1B knockdown. Scale bar = 20 μm. **d** The images of lung of nude mice in five treatment groups. Black arrowheads indicate the lung metastatic nodules stained by hematoxylin and eosin. Scale bar = 20 μm. **e** Representative phase-contrast images of circANKS1B-overexpressing MCF-7 cells and circANKS1B knockdown MDA-MB-231 cells. Scale bar = 20 μm. **f-g** Immunoblot analysis of E-cadherin, Vimentin and Fibronectin protein expression in circANKS1B-overexpressing MCF-7/T47D cells and circANKS1B silencing MDA-MB-231/BT549 cells. GAPDH was used as a loading control. Data were represented as means ± S.D. of at least three independent experiments. *p < 0.05, **p < 0.01, ***p < 0.001

To determine the biological function of circANKS1B, we first examined the expression levels of circANKS1B in breast cancer cell lines. In agreement with the results from breast cancer tissues, circANKS1B was significantly up-regulated in TNBC cell lines compared with non-TNBC cell lines (Fig. 2a). We then designed two siRNAs targeting the junction sites of circANKS1B to disrupt circANKS1B expression in MDA-MB-231 cells, qRT-PCR results showed that the expression of circANKS1B, but not its linear isoform, was specifically silenced by these two siRNAs (Additional file 1: Figure S3A), thus these two sequences were used to establish lentiviral-mediated stable circANKS1B-sliencing cell lines. In addition, the circANKS1B-expressing vector was also constructed to overexpress circANKS1B in MCF-7 cells, and we verified it could specifically increase circANKS1B expression, but not that of the un-spliced precursor (Additional file 1: Figure S3B). Ectopic expression of circANKS1B promoted, but silencing of circANKS1B inhibited, the migratory and invasive capabilities of breast cancer cells, as demonstrated by wound healing, transwell migration and invasion assays (Fig. 2b-c). Similar results were also observed in T47D and BT549 cells (Additional file 1: Figure S5A). However, circANKS1B overexpression or knockdown had no effect on cell growth and apoptosis of breast cancer (Additional file 1: Figure S4A-E). To exclude this possibility that metastasis-promoting effect is caused by linear ANKS1B, we designed two siRNAs (one siRNA targeting sequence only in linear ANKS1B, another targeting the common exon sequence of both circANKS1B and linear ANKS1B) and further confirmed their respective specific interference efficiency by qRT-PCR (Additional file 1: Figure S6A). The results showed that the migratory and invasive capabilities were dramatically reduced only in si-both, but not si-ANKS1B transfected breast cancer cells (Additional file 1: Figure S6B-C), suggesting that circANKS1B, but not linear ANKS1B, is crucial for breast cancer migration and invasion.

In order to evaluate whether circANKS1B can promote metastasis in vivo, the lung metastasis models were established by intravenously injecting MCF-7 or MDA-MB-231 cells into nude mice (8 mice in each group). Six weeks after inoculation, all mice were euthanized and lung metastatic nodules were counted. As shown in Fig. 2d, more and less metastatic nodules on the lung surfaces were respectively observed in circANKS1B overexpression and knockdown groups compared with their respective control groups, indicating that circANKS1B enhances tumor metastasis in vivo, which is consistent with our findings in vitro and in clinic.

In addition, we found that the morphology of MCF-7 cells with circANKS1B overexpression have been changed from a cobblestone shape to a fibroblast-like shape (Fig. 2e). In contrast, MDA-MB-231 cells with circANKS1B silencing showed a more epithelial morphology than that of control cells (Fig. 2e). These reveal that circANKS1B may participate in the EMT process. As expected, immunoblot analysis showed overexpression of circANKS1B decreased E-cadherin (epithelial cell marker) expression, whereas increased Vimentin and Fibronectin (mesenchymal cell markers) expression in MCF-7 and T47D cells, while circANKS1B knockdown reversed this phenomenon in MDA-MB-231 and BT549 cells (Fig. 2f-g). And these results were also substantiated by immunofluorescence analysis (Additional file 1: Figure S5B). Altogether, these findings implicate that circANKS1B promotes invasion and metastasis by regulating the EMT program in breast cancer.

### CircANKS1B serves as a sponge for miR-148a-3p and miR-152-3p in breast cancer

**Fig. 3:**
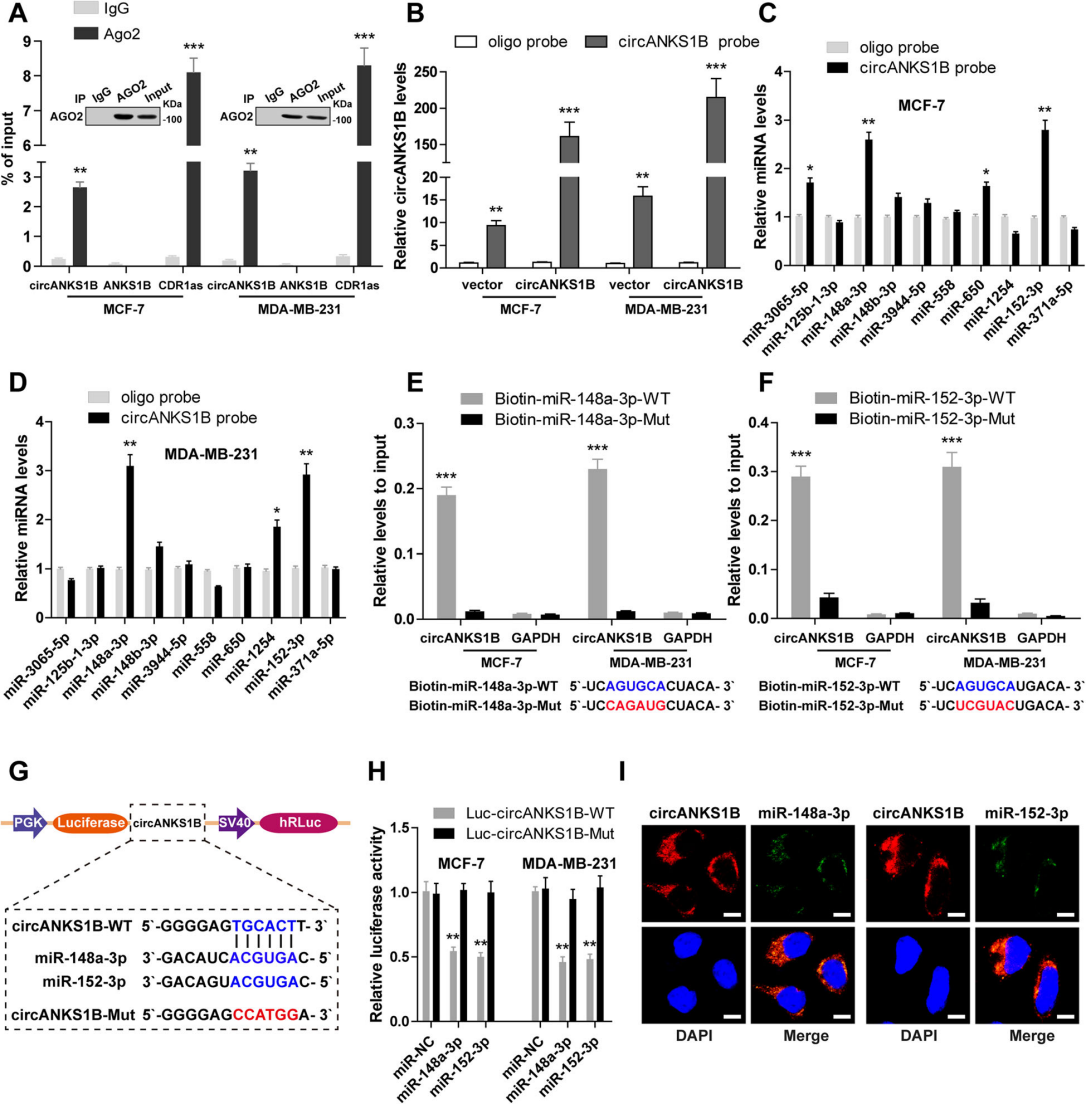
CircANKS1B functions as a sponge for miR-148a-3p and miR-152-3p in breast cancer. **a** RIP analysis of circANKS1B, linear ANKS1B and CDR1as using anti-AGO2 antibody in MCF-7 and MDA-MB-231 cells. CDR1as, which has been validated to bind to AGO2 protein, was used as a positive control. **b** Biotinylated-circANKS1B pull-down assays were performed to test the specificity of circANKS1B probe. **c-d** The top ten candidate miRNAs predicted by CircNet database were showed and their expression levels were measured by qRT-PCR after the biotinylated-circANKS1B pull-down assays in MCF-7 and MDA-MB-231 cells. **e-f** Wild-type (WT) or mutant (Mut) biotinylated-miR-148a/152-3p mimics were transfected into circANKS1B-overexpressing MCF-7 and MDA-MB-231 cells, after streptavidin capture, circANKS1B expression levels were determined by qRT-PCR. **g** Schematic of luciferase reporter vectors containing wild-type (WT) or mutant (Mut) putative miR-148a/152-3p binding sites of circANKS1B. **h** Wild-type (WT) or mutant (Mut) circANKS1B luciferase reporter vector was co-transfected with miR-148a/152-3p mimics or control mimics into MCF-7 and MDA-MB-231 cells, after 48 h, the luciferase activities were assessed. **i** FISH assays were conducted to determine the co-localization between circANKS1B and miR-148a-3p or miR-152-3p in breast cancer cells. Scale bar = 10 μm. Data were represented as means ± S.D. of at least three independent experiments. *p < 0.05, **p < 0.01, ***p < 0.001

To dissect the underlying mechanism by which circANKS1B induces EMT to promote breast cancer metastasis, we mainly focus on “miRNA sponges” because of circANKS1B is stable and located in the cytoplasm. It is well-known that miRNAs degrade mRNA and inhibit translation in an AGO2-dependent manner via directly binding to their targets [29], we thus performed RIP assay in MCF-7 and MDA-MB-231 cells. The results showed that CDR1as (a circular RNA has been reported to abundantly bind to AGO2) [17] and circANKS1B, but not linear ANKS1B, were enriched in AGO2 immunoprecipitates (Fig. 3a), indicating that circANKS1B had miRNA-related functions. We then selected the top ten candidate miRNAs (miR-3065-5p, miR-125b-1-3p, miR-148a-3p, miR-148b-3p, miR-3944-5p, miR-558, miR-650, miR-1254, miR-152-3p, and miR-371a-5p) predicted by CircNet database (http://circnet.mbc.nctu.edu.tw/) to test which miRNAs potentially interacted with circANKS1B. A biotinylated circANKS1B probe was designed and validated to specifically pull down circANKS1B in MCF-7 and MDA-MB-231 cells (Fig. 3b). RNA pull-down assays showed that miR-148a-3p and miR-152-3p were abundantly pulled down by circANKS1B probe compared with oligonucleotide probe in both MCF-7 and MDA-MB-231 cells (Fig. 3c-d). To further confirm the interactions between them, the wild-type or mutant biotinylated miR-148a-3p and miR-152-3p mimics were respectively transfected into circANKS1B-overexpressing MCF-7 and MDA-MB-231 cells, qRT-PCR results showed wild-type miR-148a-3p and miR-152-3p mimics captured more circANKS1B than their respective mutant mimics(Fig. 3e-f). In addition, we also conducted luciferase reporter assays and revealed that miR-148a-3p or miR-152-3p overexpression reduced the luciferase activity of a wild-type reporter by at least 50%, whereas had no effect on the mutant one (Fig. 3g-h). Moreover, FISH analysis indicated that circANKS1B was co-localized with miR-148a-3p or miR-152-3p in the cytoplasm (Fig. 3i). Next, we found that circANKS1B did not affect the expression of miR-148a-3p and miR-152-3p (Additional file 1: Figure S7A-B), and the expression level of circANKS1B displayed a slight change after overexpression of miR-148a-3p or miR-152-3p (Additional file 1: Figure S7C), suggesting that circANKS1B and miR-148a/152-3p may not be digested by each other. Overall, these results strongly support the idea that circANKS1B functions as miRNA sponges and demonstrate that miR-148a-3p and miR-152-3p are the circANKS1B-associated miRNAs in breast cancer.

### CircANKS1B promotes breast cancer invasion and metastasis by the miR-148a/152-3p-USF1 pathway

**Fig. 4:**
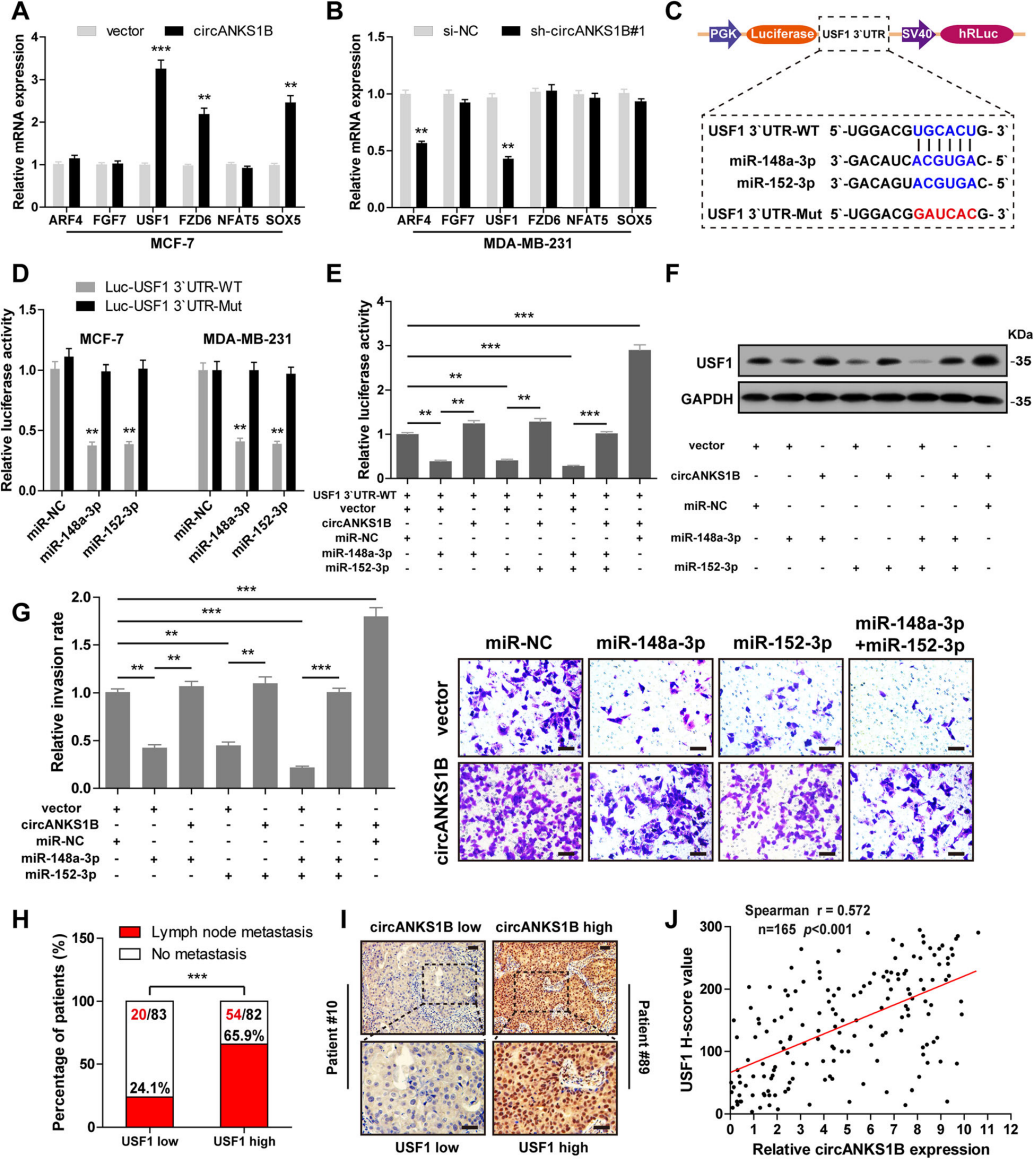
CircANKS1B promotes breast cancer invasion and metastasis by sponge activity of miR-148a-3p and miR-152-3p and up-regulation of USF1.**a-b** qRT-PCR analysis of ARF4, FGF7, USF1, FZD6, NFAT5 and SOX5 in circANKS1B-overexpressing MCF-7 cells and circANKS1B knockdown MDA-MB-231 cells. **c** Schematic of luciferase reporter vectors containing wild-type (WT) or mutant (Mut) putative miR-148a/152-3p binding sites of USF1 3′ UTR. **d** Wild-type or mutant USF1 3′ UTR luciferase reporter vector was co-transfected with miR-148a/152-3p mimics or control mimics into MCF-7 and MDA-MB-231 cells, after 48 h, the luciferase activities were measured. **e** The relative luciferase activities were analyzed after co-transfection with circANKS1B or control vector, miR-148a/152-3p mimics or control mimics and wild-type (WT) USF1 3′ UTR luciferase reporter vectors. **f** Immunoblot analysis of USF1 protein expression after co-transfection with circANKS1B or control vector and miR-148a/152-3p mimics or control mimics. GAPDH was used as a loading control. **g** Transwell invasion assay was performed after co-transfection with circANKS1B or control vector and miR-148a/152-3p mimics or control mimics. **h** Analysis of USF1 expression in breast cancer patients with or without lymph node metastasis. Using median USF1 H-score value as cutoff. **i** Representative IHC images of low (patient # 10) and high (patient # 89) USF1 expression in breast cancer tissues. Scale bar = 20 μm. **j** A strong correlation between circANKS1B and USF1 expression in breast cancer tissues analyzed by Spearman correlation coefficients (*r* = 0.572, *n* = 165, *p* < 0.001). Data were represented as means ± S.D. of at least three independent experiments. **p < 0.01, ***p < 0.001

Previous studies have reported that miR-148/152 family suppressed tumor metastasis by inhibiting the expression levels of their target oncogenes [30]. Hence, we hypothesize that circANKS1B promotes breast cancer metastasis probably by protecting these oncogenes from down-regulation by miR-148a/152-3p. Fourteen metastasis-related miR-148a/152-3p targets were selected using miRWalk 2.0 database (http://zmf.umm.uni-heidelberg.de/apps/zmf/mirwalk2/), which integrates data of ten prediction programs (miRanda, DIANA-mT, miRDB, miRWalk, RNAhybrid, PICTAR4, PICTAR5, PITA, RNA22 and TargetScan) (Additional file 1: Figure S8A). Among them, six genes (ARF4, FGF7, USF1, FZD6, NFAT5 and SOX5) were consistently down-regulated after forced expression of miR-148a-3p or miR-152-3p (Additional file 1: Figure S8B). Next, we evaluated the expression of these six genes after circANKS1B overexpression or knockdown by qRT-PCR. The results showed that overexpression of circANKS1B in MCF-7 cells significantly increased USF1, FZD6 and SOX5 expression (Fig. 4a), and silencing of circANKS1B in MDA-MB-231 significantly decreased ARF4 and USF1 expression (Fig. 4b). We then focused on USF1 to further study due to it exhibited consistent and significant changes.

The luciferase reporter assays were carried out to evaluate whether miR-148a-3p and miR-152-3p could directly bind to USF1 3′ UTR in MCF-7 and MDA-MB-231 cells (Fig. 4c), the results showed that overexpression of miR-148a-3p or miR-152-3p dramatically decreased the luciferase activity of a wild-type 3’ UTR of USF1 reporter but not the mutant one (Fig. 4d). However, these reduction effects could be rescued by overexpressing circANKS1B (Fig. 4e). Consistently, the inhibition in USF1 protein expression caused by miR-148a-3p or (and) miR-152-3p overexpression could also be abolished by ectopic expression of circANKS1B (Fig. 4f). Functionally, transwell invasion assay revealed that overexpression of miR-148a-3p or (and) miR-152-3p remarkably suppressed the invasive capacity of breast cancer cells, and the suppression could be blocked by circANKS1B overexpression (Fig. 4g). These verify our hypothesis that circANKS1B protects USF1 from down-regulation by miR-148a/152-3p. In addition, we explored the role of USF1 in breast cancer, and IHC results showed that USF1 was significantly up-regulated in breast cancer tissues, particularly in TNBC (Additional file 1: Figure S9A), which was also confirmed in TCGA database (Additional file 1: Fig. S9B). Moreover, USF1 was positively associated with lymph node metastasis (*p* < 0.001) (Fig. 4h) and the survival data of breast cancer patients from KM-plotter (http://kmplot.com/analysis/) indicated that higher USF1 expression had significantly worse overall survival (*p* < 0.001) and distant metastasis-free survival (*p* = 0.013) (Additional file 1: Figure S9C-D). Importantly, circANKS1B was positively correlated with USF1 in breast cancer tissues (*r* = 0.572, *n* = 165, *p* < 0.001) (Fig. 4i-j). Collectively, these above results suggest that circANKS1B exerts its pro-metastasis effect by positively regulating USF1 via sponge activity of miR-148a-3p and miR-152-3p in breast cancer.

### TGF-β1 is a direct transcriptional target of USF1 in breast cancer

**Fig. 5:**
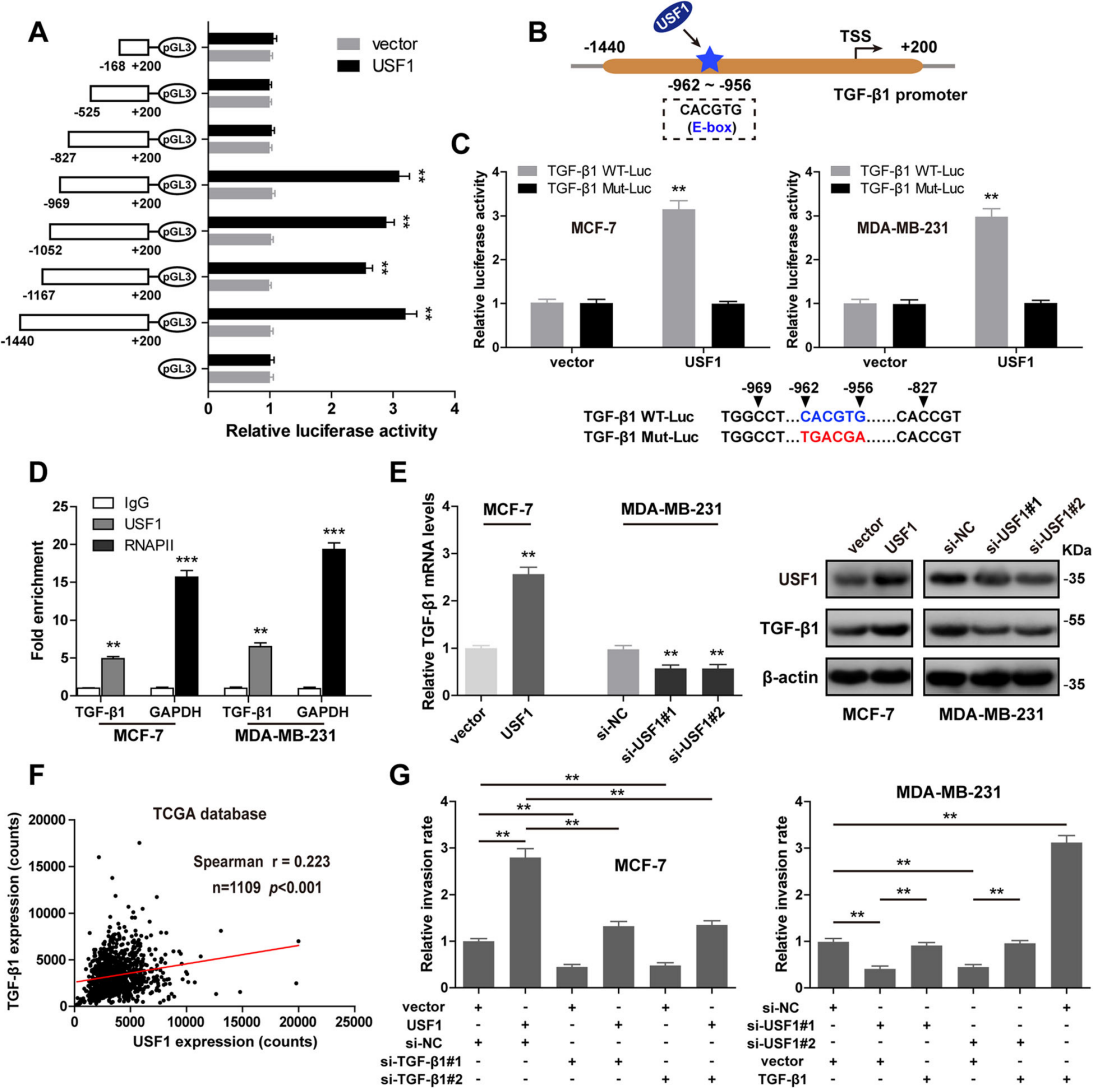
USF1 transcriptionally elevates TGF-β1 expression in breast cancer. **a** The relative luciferase activities were analyzed after co-transfection with various TGF-β1 promoter reporters or the pGL3-basic vector and USF1 or control vector. **b** Schematic of the location of E-box motif bound by USF1 in TGF-β1 promoter region. **c** Wild-type (WT) or mutant (Mut) TGF-β1 luciferase reporter vector was co-transfected with USF1 or control vector into MCF-7 and MDA-MB-231 cells, after 48 h of co-transfection, the luciferase activities were tested. **d** ChIP-qPCR analysis of USF1 binding to the TGF-β1 promoter region in MCF-7 and MDA-MB-231 cells. RNA polymerase II (RNAPII) was used as a positive control. **e** qRT-PCR (left) and immunoblot (right) analysis of TGF-β1 mRNA expression in USF1-overexpressing MCF-7 cells and USF1 knockdown MDA-MB-231 cells. β-actin was used as a loading control. **f** The correlation between USF1 and TGF-β1 expression in breast cancer tissues from TCGA database was analyzed by Spearman correlation coefficients (*r* = 0.223, *n* = 1109, *p* < 0.001). **g** Transwell invasion assays were carried out in MCF-7 cells co-transfected with USF1 or control vector and si-TGF-β1 or si-NC (left) and MDA-MB-231 cells co-transfected with si-USF1 or si-NC and TGF-β1 or vector (right). Data were represented as means ± S.D. of at least three independent experiments. **p < 0.01, ***p < 0.001

USF1, a transcription factor, belongs to the basic helix-loop-helix leucine zipper (bHLH-LZ) family which regulates different genes expression by binding to consensus sequence of E-box (CANNTG) in their promoter regions [31]. A previous study showed that USF1 could bind to the promoter of murine TGF-β1 [32], which was a well-known major driver of EMT [7]. Thus, we presume that USF1 contributes to breast cancer metastasis by regulating TGF-β1-mediated EMT process. In order to verify this hypothesis, we first test whether USF1 can also bind to human TGF-β1 promoter. A series of TGF-β1 promoter reporter constructs were generated, including the full-length TGF-β1 promoter construct and six truncation constructs. Luciferase reporter assay showed that the region of the TGF-β1 promoter from − 969 to − 827 bp was critical for USF1-mediated transcriptional regulation of TGF-β1 (Fig. 5a). Based on the binding sites predicted by Jaspar (http://jaspardev.genereg.net/) and ConSite (http://consite.genereg.net/), we found an E-box motif (CACGTG) at position − 962/− 956 of TGF-β1 promoter (Fig. 5b). To investigate whether USF1 regulates TGF-β1 promoter activity by binding to this motif, luciferase reporter assay was performed and showed that USF1 overexpression increased the luciferase activity of the reporter containing wild-type E-box motif of TGF-β1 promoter, while did not affect that of the mutant reporter in MCF-7 and MDA-MB-231 cells (Fig. 5c). And ChIP-qPCR analysis revealed that the promoter sequences of TGF-β1 were specifically enriched by anti-USF1 antibody, but not by negative control antibody IgG (Fig. 5d). These indicate USF1 can directly bind to TGF-β1 promoter to increase its transcriptional activity in breast cancer cells. Next, we found that forced expression of USF1 increased, but knockdown of USF1 decreased, both mRNA and protein expression levels of TGF-β1 (Fig. 5e). And USF1 was positively correlated with TGF-β1 in breast cancer tissues from the TCGA database (*r* = 0.223, *n* = 1109, *p* < 0.001) (Fig. 5f). Functionally, transwell invasion assay showed that USF1 overexpression significantly increased the invasive capacity of MCF-7 cells, and the increase could be retarded by silencing of TGF-β1. In contrast, knockdown of USF1 significantly reduced the invasive capacity of MDA-MB-231 cells, and the reduction could be rescued after ectopic expression of TGF-β1 (Fig. 5g) (Additional file 1: Figure S10A-B). These above data suggest that USF1 enhances breast cancer invasion and metastasis by transcriptionally elevating TGF-β1 expression.

### ESRP1 promotes the generation of circANKS1B and it is also a transcriptional target of USF1 in breast cancer

**Fig. 6:**
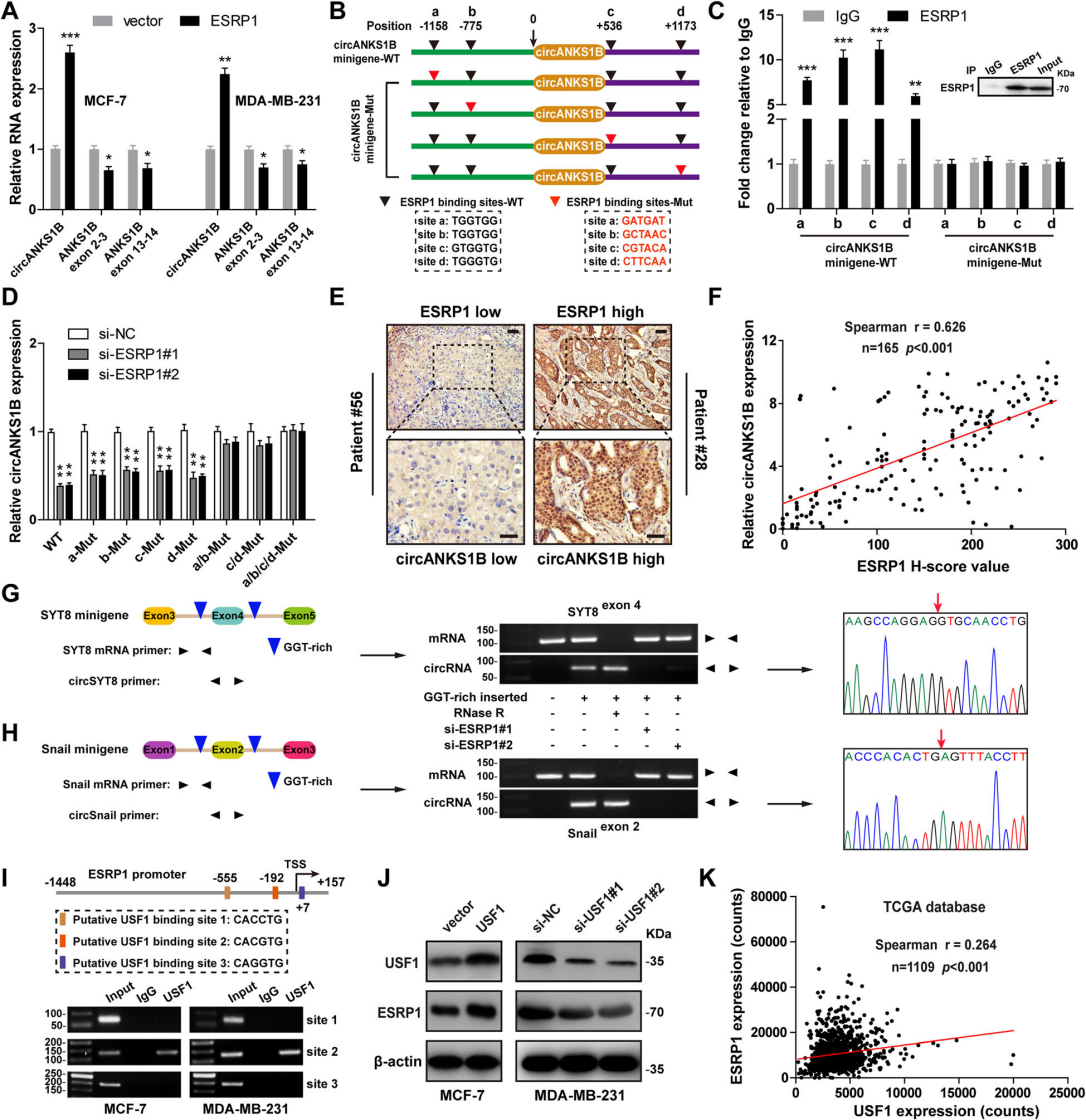
ESRP1 promotes circANKS1B formation and it is also a target of USF1 in breast cancer. **a** qRT-PCR analysis of circANKS1B and linear ANKS1B in MCF-7 and MDA-MB-231 cells with ESRP1 overexpression. **b** Schematic of circANKS1B minigene with four wild-type (WT) or mutant (Mut) ESRP1 binding sites. **c** RIP analysis of ESRP1-binding to wild-type (WT) or mutant (Mut) circANKS1B minigene using an antibody against ESRP1. **d** qRT-PCR analysis of circANKS1B after co-transfection with si-ESRP1 or si-NC and wild-type (WT) or various mutant (Mut) circANKS1B minigenes. **e** Representative IHC images of low (patient # 56) and high (patient # 28) ESRP1 expression in breast cancer tissues. Scale bar = 20 μm. **f** A strong correlation between ESRP1 and circANKS1B expression in breast cancer tissues assessed by Spearman correlation coefficients (*r* = 0.626, n = 165, *p* < 0.001). **g-h** Schematic of insertion of ESRP1 binding sites (GGT-rich) and locations of primers used for segments of two genes (SYT8 and Snail) devoid of circRNAs (left). RT-PCR analysis of circSYT8, linear SYT8, circSnail and linear Snail in MDA-MB-231 cells transfected with SYT8 or Snail minigene, with or without si-ESRP1 or treatment with RNase R (middle). The formation of circSYT8 and circSnail through back-splicing was confirmed by Sanger sequencing, red arrow indicates the junction site (right). **i** Schematic of three putative USF1 binding sites in ESRP1 promoter region (above). ChIP-PCR assays were performed to determine which putative USF1 binding site was bound by USF1 in MCF-7 and MDA-MB-231 cells (below), IgG was used as a negative control. **j** Immunoblot analysis of USF1 and ESRP1 in USF1-overexpressing MCF-7 cells and USF1 knockdown MDA-MB-231 cells. β-actin was used as a loading control. **k** The correlation between USF1 and ESRP1 expression in breast cancer tissues from TCGA database was determined by Spearman correlation coefficients (*r* = 0.264, n = 1109, *p* < 0.001). Data were represented as means ± S.D. of at least three independent experiments. *p < 0.05, **p < 0.01, ***p < 0.001

We next interrogate the mechanism by which circANKS1B is generated. Recent studies showed that splicing factors were capable of regulating cell-type-specific circRNA expression [16] and some EMT-associated splicing factors have also been identified [15]. A series of siRNAs targeting these EMT-associated splicing factors were designed to assess which splicing factor potentially participated in circANKS1B generation. The qRT-PCR results showed that silencing of ESRP1 noticeably decreased circANKS1B expression in MDA-MB-231 cells (Additional file 1: Fig. S11A). Conversely, ESRP1 overexpression significantly increased circANKS1B expression in both MCF-7 and MDA-MB-231 cells, while reduced the expression of linear ANKS1B isoform (Fig. 6a), suggesting ESRP1 regulates the expression of circANKS1B. Notably, knockdown of ESRP1 also inhibited breast cancer invasion and the effect could be largely rescued by circANKS1B overexpression, as showed by transwell invasion assay (Additional file 1: Figure S11B). It has been reported that splicing factors promoted the formation of circRNA by directly binding to the sequences on the introns adjacent to the circRNA-forming exons [15]. To test whether ESRP1-binding sequences (GGT-rich) [33] on the flanking introns are required for circANKS1B biogenesis, we first identify four putative ESRP1 binding sites, including two are located upstream and two are located downstream of the circANKS1B-forming splice sites, as shown in Fig. 6b. We then constructed the wild-type and mutant circANKS1B minigenes to conduct RIP assay. The results indicated that ESRP1 could bind to these four putative wild-type binding sites on the flanking introns, but not the mutant sites (Fig. 6c). Furthermore, we found that compared with si-NC, ESRP1 knockdown dramatically decreased circANKS1B production in breast cancer cells transfected with wild-type or individually mutation circANKS1B minigenes, whereas had little or no effect on circANKS1B production in cells transfected with a/b or (and) c/d mutation minigenes (Fig. 6d), suggesting that these four motifs on flanking introns are necessary for ESRP1-mediated circANKS1B biogenesis. Clinically, we found that ESRP1 expression was positively associated with circANKS1B expression in breast cancer tissues (*r* = 0.626, *n* = 165, *p* < 0.001) (Fig. 6e-f). And breast cancer patients with high ESRP1 expression displayed poor overall survival (*p* < 0.001) and distant metastasis-free survival (*p* < 0.001) (Additional file 1: Figure S11C-D).

We then ask whether exons that do not normally give rise to circRNAs could be made competent to generate circRNAs by insertion of ESRP1-binding motifs into the flanking introns. We chose two genes (SYT8 and Snail) to construct minigenes due to both of them could not form circRNAs according to the data from circBase (http://www.circbase.org/). As shown in Fig. 6g-h, the minigenes contained 3 exons from each of these two genes with or without inserting ESRP1-binding motifs (GGT-rich) into both introns flanking the central exon and were transfected into MDA-MB-231 cells. The results of RT-PCR showed that the inserted ESRP1-binding motifs minigenes, but not the unmodified minigenes, were capable of generating circRNAs, which were also confirmed by Sanger sequencing and their resistance to RNase R (Fig. 6g-h), and the production of circRNAs was severely blocked after silencing of ESRP1, revealing that insertion of ESRP1-binding motifs into the flanking introns is sufficient to induce circRNAs generation from transcripts that are normally linearly spliced. These data collectively indicate that ESRP1 plays a vital role in circANKS1B biogenesis in breast cancer.

Intriguingly, three E-box motifs are found in ESRP1 promoter region (Fig. 6i), we then hypothesize that USF1 can also transcriptionally regulate ESRP1 expression. To test this hypothesis, ChIP assays were performed and RT-PCR results showed that USF1 could only bound to the putative binding site 2 (CACGTG) at position − 192/− 187 of ESRP1 promoter in MCF-7 and MDA-MB-231 cells (Fig. 6i). Further, we found that overexpression of USF1 increased, knockdown of USF1 decreased, the expression of ESRP1 (Fig. 6j). And a positive correlation between USF1 and ESRP1 expression was observed in breast cancer tissues from TCGA database (*r* = 0.264, *n* = 1109, *p* < 0.001) (Fig. 6k). These suggest that ESRP1 is also a direct transcriptional target of USF1 in breast cancer.

### The ESRP1/circANKS1B/miR-148a/152-3p/USF1 feedback loop promotes cell invasion and metastasis via inducing TGF-β1-mediated EMT in breast cancer

**Fig. 7:**
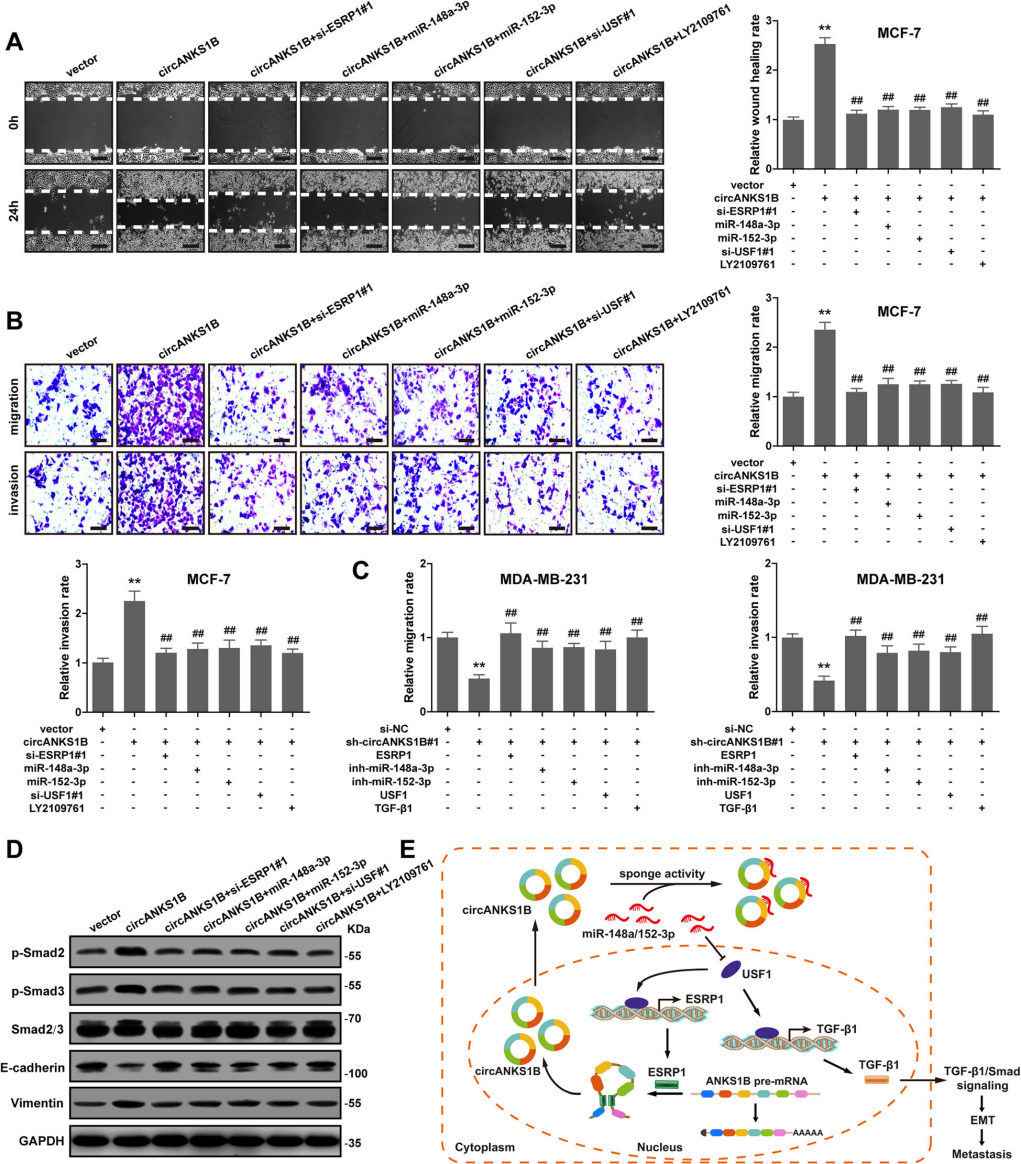
The ESRP1/circANKS1B/miR-148a/152-3p/USF1 feedback loop promotes breast cancer invasion and metastasis via inducing TGF-β1/Smad-mediated EMT. **a-b** Wound healing, transwell migration and invasion assays for circANKS1B-overexpressing MCF-7 cells transfected with si-ESRP1, si-USF1 or miR-148a/152-3p mimics or treated with LY2109761 at a final concentration of 10 μm. Representative images are shown at 0 and 24 h after gap creation. Scale bar = 20 μm. **c** Transwell migration and invasion assays for circANKS1B silencing MDA-MB-231 cells transfected with ESRP1, USF1 or TGF-β1 vector, or miR-148a/152-3p inhibitors. **d** Immunoblot analysis of p-Smad2, p-Smad3, Smad2/3, E-cadherin and Vimentin in circANKS1B-overexpressing MCF-7 cells transfected with si-ESRP1, si-USF1 or miR-148a/152-3p mimics or treated with LY2109761 at a final concentration of 10 μM. GAPDH was used as a loading control. **e** The illustration summarizes our findings. CircANKS1B, as miR-148a-3p and miR-152-3p sponge, increases USF1 expression by eliminating miR-148a/152-3p-mediated repression of USF1, and then, USF1 can respectively transcriptionally up-regulate ESRP1 and TGF-β1 expression via directly binding to the E-box motifs in their promoter regions. Subsequently, ESRP1 promotes circANKS1B generation, and TGF-β1 activates its downstream Smad signaling to induce EMT, thereby enhancing breast cancer invasion and metastasis. Data were represented as means ± S.D. of at least three independent experiments. **p < 0.01 versus control group, ^**##**^*p* < 0.01 versus circANKS1B overexpression or knockdown group

Next, we found that the increased migratory and invasive capabilities of MCF-7 cells caused by circANKS1B overexpression could be extensively retarded by ESRP1 or USF1 knockdown or miR-148a/152-3p overexpression, as well as by treatment with LY2109761 (TGF-β receptor type I/II inhibitor), as demonstrated by wound healing, transwell migration and invasion assays (Fig. 7a-b). On the contrary, disruption of circANKS1B inhibited MDA-MB-231 cells migration and invasion, and the inhibition could be largely blocked by forced expression of ESRP1, USF1 or TGF-β1, as well as by silencing of miR-148a-3p or miR-152-3p (Fig. 7c). As expected, the immunoblot analysis showed that circANKS1B overexpression remarkably elevated expression of p-Smad2, p-Smad3 and Vimentin, while dramatically reduced E-cadherin expression, and these effects were hindered after ESRP1 or USF1 disruption or ectopic expression of miR-148a/152-3p, or adding LY2109761 (Fig. 7d). These results indicate that the ESRP1/circANKS1B/miR-148a/152-3p/USF1 feedback loop does exist in breast cancer and it can induce EMT by activating TGF-β1 signaling to promote breast cancer metastasis (Fig. 7e).

## Discussion

In the present study, we identified a large amount of circRNAs by RNA-seq. And we then characterized one of the most differentially expressed circRNAs, circANKS1B, which was highly associated with breast cancer invasion and metastasis and poor prognosis. Functionally, circANKS1B promoted breast cancer cell invasion and metastasis without affecting cell proliferation and apoptosis. Mechanistically, circANKS1B could up-regulate USF1 expression by sponge activity of miR-148a-3p and miR-152-3p. Further, USF1 transcriptionally elevated ESRP1 and TGF-β1 expression through directly binding to their promoters, thereby activating TGF-β1 signaling to enhance EMT and metastasis. Besides, we found that ESRP1 increased circANKS1B production via interaction with its flanking introns. Thus, our findings identify a novel feedback loop that promotes breast cancer metastasis, which advance the understanding of molecular mechanism involved in the metastasis of breast cancer.

Accumulating evidence shows that circRNAs are abundant, stable and highly conserved in eukaryotes with gene-regulatory potency [12, 17]. Here, we also identify a large number of circRNAs (69,815) and most of them are generated from precursor mRNAs by exon circularization. Amino acid sequence alignment shows that the similarity of human circANKS1B with homolog in *Mus musculus* is 88% (data not shown), suggesting circANKS1B is a well-conserved gene. The abundance, stability and specific expression patterns of circRNAs allowing circRNAs to be the promising potential cancer biomarkers. Up to now, many circRNAs have been identified as diagnostic and prognostic biomarkers in human malignancy, such as colorectal cancer (CiRS-7 and circHIPK3) [21, 34], gastric cancer (circPVT1 and hsa_circ_0000096) [35, 36], hepatocellular carcinoma (circMTO1 and circSMARCA5) [20, 37] and bladder cancer (circMYLK and circITCH) [19, 38]. In this study, we found that breast cancer patients with higher circANKS1B expression displayed significantly worse overall survival, and high circANKS1B was an independent factor for poor outcome, as demonstrated by Cox proportional hazards model. These indicate that circANKS1B may be a promising prognostic biomarker in breast cancer.

It has been reported that circRNAs, like LncRNAs, exerted diverse biological functions by acting as miRNA sponges [39–41]. Of note, due to the covalently closed structure of circRNA, it may maintain the miRNA-regulatory function for a longer period of time than LncRNA. Herein, using various assays, we found that circANKS1B promoted breast cancer invasion and metastasis, mainly through interaction with miR-148a-3p and miR-152-3p. The miR-148/152 family consists of miR-148a-3p, miR-148b-3p and miR-152-3p, is proposed to be potential metastasis suppressors in many cancers, including breast cancer [42]. Mature miR-148/152 family shares similar seed sequence, which is a key region for regulating their targets [30]. However, the RNA pull-down results showed that miR-148a-3p and miR-152-3p, but not miR-148b-3p, were abundantly pulled down by circANKS1B probe in both MCF-7 and MDA-MB-231 cells, revealing that miR-148b-3p might not be involved in circANKS1B-mediated metastasis-promoting process in breast cancer, this was also confirmed by the rescue experiment that inhibition of miR-148b-3p could not rescue the decreased migratory and invasive capabilities of breast cancer cells caused by silencing of circANKS1B (Additional file 1: Figure S12A-B). By a series of screenings and validations, we identified that USF1, the common target of miR-148a-3p and miR-152-3p, participated in circANKS1B-mediated pro-metastasis process in breast cancer. USF1 is a transcription factor and it can regulate the expression of different genes by binding to the E-box motifs (CANNTG) in their promoter regions [31]. As the previous study reported that USF1 could bind to murine TGF-β1 promoter [32], we then wonder whether this phenomenon also occurs on human TGF-β1 promoter. ChIP-qPCR and luciferase reporter assays clearly showed that USF1 could directly bind to human TGF-β1 promoter to transcriptionally up-regulate TGF-β1 expression. TGF-β1 is a well-known driver of EMT, which is critical for breast cancer metastasis [7]. Therefore, these data show that circANKS1B, as miR-148a-3p and miR-152-3p sponge, relieves the miR-148a/152-3p-mediated inhibition of USF1 which subsequently transcriptionally elevates TGF-β1 to induce EMT, thus promoting breast cancer invasion and metastasis, supporting the notion that circANKS1B is capable of functioning as a miRNA sponge to modulate gene expression in breast cancer.

Recent studies showed that splicing factors played crucial roles in cell-type-specific circRNA formation [15, 16]. Here, we identified a splicing factor, ESRP1, was involved in circANKS1B biogenesis in breast cancer. We found that ESRP1 promoted circANKS1B formation by interaction with “GGT-rich” motifs on upstream and downstream of the introns flanking circANKS1B-forming exons. Silencing of ESRP1 decreased circANKS1B expression by about 55%, suggesting that other splicing factors might participate in circANKS1B production. As shown in Additional file 1: Figure S10A, knockdown of ESRP2, a close family member of ESRP1 that recognizes the similar binding motif, partially reduced circANKS1B expression, and knockdown of QKI, a splicing factor was proposed to regulate over one-third of EMT-related circRNAs expression, could decreased circANKS1B expression by about 25%, implying that circRNAs biogenesis may be simultaneously controlled by multiple splicing factors. In addition, we found that ESRP1 disruption increased linear ANKS1B expression, this may be explained by the competition between circRNA and its linear isoform during pre-mRNA splicing [16]. Importantly, mutation of the ESRP1-binding motifs on the flanking introns dramatically decreased circANKS1B formation, whereas exons that do not normally give rise to circRNAs could be capable of generating circRNAs by insertion of ESRP1-binding motifs into the flanking introns in breast cancer cells. And a recent study demonstrated that ESRP1 could also promote the generation of circBIRC6 in human embryonic stem cells [25]. These indicate that ESRP1 may regulate multiple circRNAs and further studies will be warranted to explore the role of ESRP1-mediated circRNA biogenesis in other diseases. Moreover, we showed that ESRP1 was also a direct transcriptional target of USF1, providing evidence to further support the idea that the ESRP1-circANKS1B axis is a metastasis-associated regulatory pathway.

Although the metastasis-promoting effect of circANKS1B in breast cancer was illustrated in our study, owing to the limited breast cancer tissues we screened initially, we do not rule out this possibility that there may be other key dysregulated circRNAs which are also involved in breast cancer metastasis, as well as some other pathological processes such as proliferation or apoptosis. Therefore, the dysregulated circRNAs in breast cancer still need further elucidation.

## Conclusions

In summary, our study convincingly demonstrates that the newly identified ESRP1/circANKS1B/miR-148a/152-3p/USF1 regulatory circuit can induce EMT via the activation of TGF-β1 signaling pathway, thereby contributing to breast cancer invasion and metastasis, meanwhile facilitating the development of new treatment strategy against the metastasis of breast cancer.

## Additional file

Additional file 1: **Tables S1.** Correlations between circANKS1B expression and clinical characteristics in breast cancer patients (*n* = 165). **Tables S2.** Univariate and multivariate overall survival analysis of prognostic factors for breast cancer patients (*n* = 165). **Tables S3.** Primers and RNA sequences used in this study. **Figure S1.** (A) Genomic origin of circRNAs (*n* = 69,815) identified in human breast tissues. 87% were derived from exons, and the others were derived from introns, intergenic region and 3′ or 5′ UTR, etc. (B) The length distribution for circRNAs (*n* = 69,815) identified in human breast tissues. Most of the circRNAs are less than 1,300 nucleotides (nt) in length. **Figure S2.** (A-D) qRT-PCR analysis of the screened top ten most increased and decreased circRNAs in twenty pairs of TNBC and adjacent normal tissues. Four circRNAs (circ-PGAP3, circ-THSD4, circ-CYP24A1 and circ-ACACB) were validated to be significantly dysregulated. (E-F) qRT–PCR analysis of the abundance of circANKS1B and ANKS1B mRNA in MCF-7 and MDA-MB-231 cells treated with Actinomycin D at the indicated time points. (G) qRT–PCR analysis of circANKS1B and ANKS1B mRNA after treatment with RNase R in MCF-7 and MDA-MB-231 cells. **Figure S3.** (A) Schematic of two siRNA targeting circANKS1B junction site (left). These two siRNA effectively silenced circANKS1B expression in MDA-MB-231 cells, whereas had no effect on linear ANKS1B expression (right). (B) Schematic of construction of circANKS1B overexpression vector, the sequence of the 5′-flanking intron was copied and inversely inserted the downstream of 3′-flanking intron (left). The overexpression vector effectively increased circANKS1B expression in MCF-7 cells, while did not affect its precursor expression. **Figure S4.** (A-C) CCK-8 and EdU analysis of the proliferative abilities of MCF-7 cells with circANKS1B overexpression and MDA-MB-231 cells with circANKS1B knockdown. Scale bar = 20 μm. (D) The images of tumor-bearing nude mice from the indicated treatment groups (*n* = 5 for each group) on the 49th day. (E) Annexin V-PE/7-AAD double staining analysis of apoptosis of MCF-7 cells with circANKS1B overexpression and MDA-MB-231 cells with circANKS1B knockdown. **Figure S5.** (A) Transwell migration and matrigel invasion assays for T47D cells with circANKS1B overexpression and BT549 cells with circANKS1B knockdown. Scale bar = 20 μm. (B) Immunofluorescence analysis of E-cadherin and Vimentin in circANKS1B-overexpressing MCF-7 cells and circANKS1B knockdown MDA-MB-231 cells. Scale bar = 20 μm. **Figure S6.** (A) Schematic illustration showing two targeted siRNAs. si-ANKS1B targets the ANKS1B linear transcript, si-both targets both the linear ANKS1B and circANKS1B (left). Their respective inhibitory effects were verified by qRT-PCR (right). (B-C) Wound healing, transwell migration and invasion assays for MDA-MB-231 cells transfected with si-NC, si-both or si-ANKS1B. Scale bar = 20 μm. **Figure S7.** (A-B) qRT-PCR analysis of miR-148a-3p and miR-152-3p in circANKS1B-overexpressing MCF-7 cells and circANKS1B knockdown MDA-MB-231 cells. (C) qRT-PCR analysis of circANKS1B in MCF-7 and MDA-MB-231 cells transfected with miR-148a/152-3p mimics or control mimics. **Figure S8.** (A) Schematic of the selection of these 14 metastasis-related genes targeted by miR-148a-3p and miR-152-3p. (B) qRT-PCR analysis of these 14 metastasis-related genes in MCF-7 with miR-148a-3p or miR-152-3p overexpression. Solid red and black circles indicate the genes regulated and non-regulated by miR-148a-3p or miR-152-3p, respectively. **Figure S9.** (A) IHC analysis of USF1 in normal tissues (*n* = 40) and breast cancer tissues (*n* = 165). (B) The expression of USF1 mRNA in breast cancer tissues from TCGA database. (C) The overall survival curves in breast cancer patients with low and high USF1 expression from KM-plotter database (http://kmplot.com/analysis/). (D) The distant metastasis-free survival curves in breast cancer patients with low and high USF1 expression from KM-plotter database. **Figure S10.** Immunoblot analysis of USF1 and TGF-β1 in MCF-7 (A) or MDA-MB-231 cells (B) in the indicated groups. β-actin was used as a loading control. **Figure S11.** (A) qRT-PCR analysis of circANKS1B expression in MDA-MB-231 cells transfected with the indicated siRNAs. (B) Transwell invasion assay for MDA-MB-231 cells co-transfected with si-ESRP1 or si-NC and circANKS1B or control vector. (C-D) The overall and distant metastasis-free survival curves in breast cancer patients with low and high ESRP1 expression from KM-plotter database (http://kmplot.com/analysis/). Data were represented as means ± S.D. of at least three independent experiments. **Figure S12.** (A-B) Transwell migration and invasion assays for circANKS1B silencing MDA-MB-231 cells transfected with miR-148b-3p inhibitors. Data were represented as means ± S.D. of at least three independent experiments. Scale bar = 20 μm. **p < 0.01, n.s = not significant. (DOCX 25384 kb)

## Abbreviations

3’UTR: 3’-untranslated region
cDNA: Complementary DNA
ChIP: Chromatin immunoprecipitation
CircRNA: Circular RNA
EMT: Epithelial-to-mesenchymal transition
ER: Estrogen receptor
FISH: Fluorescence in situ hybridization
gDNA: Genomic DNA
HER2: Human epidermal growth factor receptor 2
IHC: Immunohistochemistry
LncRNA: Long non-coding RNA
miRNA: MicroRNA
PR: Progesterone receptor
qRT-PCR: Quantitative reverse transcription polymerase chain reaction
RIP: RNA immunoprecipitation
TCGA: The Cancer Genome Atlas
TNBC: Triple-negative breast cancer
