# Supplementary material for: The pro-metastasis effect of circANKS1B in breast cancer
Source: Mol Cancer. 2018 Nov 19;17:160. doi: 10.1186/s12943-018-0914-x (PMC6240936; doi:10.1186/s12943-018-0914-x)
Supplement: Supplementary file 1 — Tables S1. Correlations between circANKS1B expression and clinical characteristics in breast cancer patients (n = 165). Tables S2. Univariate and multivariate overall survival analysis of prognostic factors for breast cancer patients (n = 165). Tables S3. Primers and RNA sequences used in this study. Figure S1. (A) Genomic origin of circRNAs (n = 69,815) identified in human breast tissues. 87% were derived from exons, and the others were derived from introns, intergenic region and 3′ or 5′ UTR, etc. (B) The length distribution for circRNAs (n = 69,815) identified in human breast tissues. Most of the circRNAs are less than 1,300 nucleotides (nt) in length. Figure S2. (A-D) qRT-PCR analysis of the screened top ten most increased and decreased circRNAs in twenty pairs of TNBC and adjacent normal tissues. Four circRNAs (circ-PGAP3, circ-THSD4, circ-CYP24A1 and circ-ACACB) were validated to be significantly dysregulated. (E-F) qRT–PCR analysis of the abundance of circANKS1B and ANKS1B mRNA in MCF-7 and MDA-MB-231 cells treated with Actinomycin D at the indicated time points. (G) qRT–PCR analysis of circANKS1B and ANKS1B mRNA after treatment with RNase R in MCF-7 and MDA-MB-231 cells. Figure S3. (A) Schematic of two siRNA targeting circANKS1B junction site (left). These two siRNA effectively silenced circANKS1B expression in MDA-MB-231 cells, whereas had no effect on linear ANKS1B expression (right). (B) Schematic of construction of circANKS1B overexpression vector, the sequence of the 5′-flanking intron was copied and inversely inserted the downstream of 3′-flanking intron (left). The overexpression vector effectively increased circANKS1B expression in MCF-7 cells, while did not affect its precursor expression. Figure S4. (A-C) CCK-8 and EdU analysis of the proliferative abilities of MCF-7 cells with circANKS1B overexpression and MDA-MB-231 cells with circANKS1B knockdown. Scale bar = 20 μm. (D) The images of tumor-bearing nude mice from the indicated treatment groups (n [file 12943_2018_914_MOESM1_ESM.docx]

**Table S1.** Correlations between circANKS1B expression and clinical characteristics in breast cancer patients (*n* = 165)

| Clinicopathologic parameters | Total (n=165) | circANKS1B expression ^a^ | | *p* value |
| --- | --- | --- | --- | --- |
|  |  | Low (%) | High (%) |  |
| Age (years) |  |  |  |  |
| ≤ 40 | 32 | 18 (56.3%) | 14 (43.7%) | 0.454 |
| > 40 | 133 | 65 (48.1%) | 68 (51.9%) |  |
| Menopausal status |  |  |  |  |
| Premenopausal | 93 | 45 (48.4%) | 48 (51.6%) | 0.576 |
| Postmenopausal | 72 | 38 (52.8%) | 34 (47.2%) |  |
| Tumor size (cm) |  |  |  |  |
| ≤ 2 | 119 | 56 (47.1%) | 63 (52.9%) | 0.180 |
| > 2 | 46 | 27 (58.7%) | 19 (41.3%) |  |
| Lymph node metastasis |  |  |  |  |
| Negative | 91 | 55 (60.4%) | 36 (39.6%) | **0.004^**^** |
| Positive | 74 | 28 (37.8%) | 46 (62.2%) |  |
| Histological grade |  |  |  |  |
| I | 14 | 7 (50%) | 7 (50%) | 0.158 |
| II | 113 | 62 (54.9%) | 51 (45.1%) |  |
| III | 38 | 14 (36.8%) | 24 (63.2%) |  |
| TNM stage |  |  |  |  |
| I | 38 | 25 (65.8%) | 13 (34.2%) | **0.013^*^** |
| II | 73 | 40 (54.8%) | 33 (45.2%) |  |
| III | 46 | 16 (34.8%) | 30 (65.2%) |  |
| IV | 8 | 2 (25%) | 6 (75%) |  |
| ER status |  |  |  |  |
| Negative | 59 | 27 (45.8%) | 32 (54.2%) | 0.384 |
| Positive | 106 | 56 (52.8%) | 50 (47.2%) |  |
| PR status |  |  |  |  |
| Negative | 68 | 38 (55.9%) | 30 (44.1%) | 0.230 |
| Positive | 97 | 45 (46.4%) | 52 (53.6%) |  |
| HER2 status |  |  |  |  |
| Negative | 117 | 54 (46.2%) | 63 (53.8%) | 0.096 |
| Positive | 48 | 29 (60.4%) | 19 (39.6%) |  |
| Ki-67 status |  |  |  |  |
| ≤ 14% | 39 | 21 (53.8%) | 18 (46.2%) | 0.613 |
| > 14% | 126 | 62 (49.2%) | 64 (50.8%) |  |

**^*^***p* <0.05 ; **^**^***p* <0.01 ; ^a^ Using median circANKS1B value as cutoff ;

ER = estrogen receptor, PR = progesterone receptor, HER2 = human epidermal growth factor receptor 2 ;

**Table S2.** Univariate and multivariate overall survival analysis of prognostic factors for breast cancer patients (*n* = 165)

| Clinicopathologic Parameters | Overall survival |  |
| --- | --- | --- |
|  | Univariate analysis | Multivariate analysis |
|  | HR 95%CI *p* value | HR 95%CI *p* value |
| Age | 0.74 0.37-1.58 0.614 |  |
| Menopausal status | 0.96 0.51-2.13 0.812 |  |
| Tumor size | 1.35 0.79-2.94 0.347 |  |
| Lymph node metastasis | 4.96 2.68-7.47 **< 0.001^***^** | 4.78 1.39-8.69 **0.003^**^** |
| Histological grade | 2.08 1.18-3.65 **0.046^*^** | 1.56 0.48-3.47 0.258 |
| TNM stage | 4.54 2.14-9.31 **0.002^**^** | 3.78 1.89-6.37 **0.016^*^** |
| ER status | 1.14 0.47-3.69 0.318 |  |
| PR status | 1.02 0.64-2.17 0.429 |  |
| HER2 status | 1.57 0.89-3.64 0.366 |  |
| Ki-67 status | 1.86 0.92-4.38 0.237 |  |
| circANKS1B expression ^a^ | 3.95 1.47-6.82 **0.006^**^** | 3.29 1.75-8.23 **0.008^**^** |

**^*^***p* < 0.05; **^**^***p* < 0.01; **^***^***p* < 0.001; ^a^ Using median circANKS1B value as cutoff ;

ER = estrogen receptor, PR = progesterone receptor, HER2 = human epidermal growth factor receptor 2

**Table S3.** Primers and RNA sequences used in this study

| **Name** | **Sequence** | | **Application** |
| --- | --- | --- | --- |
| Circ-MCM4-forward | CCTGGGGACAGAGTGAATGT | | circRNA validation |
| Circ-MCM4--reverse | CACACACTTGGCACTGGAAG | | circRNA validation |
| Circ-THSD4-forward | CTCGAAGTCACTGGGGACAG | | circRNA validation |
| Circ-THSD4-reverse | CATGAAATGGGAAACCATGA | | circRNA validation |
| Circ-IFI6-forward | CTGGTCTGCGATCCTGAATG | | circRNA validation |
| Circ-IFI6-reverse | TCCGTCACTAGACCGAGGCT | | circRNA validation |
| Circ-PGAP3-forward | GCTCTACCTCCAGGAAGGTCAC | | circRNA validation |
| Circ-PGAP3-reverse | TCATACTTACAGTCGTCCCGACA | | circRNA validation |
| Circ-ERBB2-forward | GCCCTGGTCACCTACAACAC | | circRNA validation |
| Circ-ERBB2-reverse | AGCATGTCCAGGTGGGTCT | | circRNA validation |
| Circ-CYP24A1-forward | CACAGACAATGAGCCTGTTGA | | circRNA validation |
| Circ-CYP24A1-reverse | TCATCACTTCCCCTGGTTTC | | circRNA validation |
| Circ-ACACB-forward | TTCGACGTCCTGCCTACTTT | | circRNA validation |
| Circ-ACACB-reverse | ACTTTGCAGTGCTCGCTTTT | | circRNA validation |
| Circ-CAPG-forward | TTTGCCCTTGAACTGCTGAT | | circRNA validation |
| Circ-CAPG-reverse | CGTTCCAGGATGTTGGACTT | | circRNA validation |
| Circ-NR3C2-forward | GGAGTCATGGAAATCACACG | | circRNA validation |
| Circ-NR3C2-reverse | GACCTTCAGGGAGACTGTGG | | circRNA validation |
| Circ-SLC9A3R1-forward | GGACAGGGAAACTGACGAGT | | circRNA validation |
| Circ-SLC9A3R1-reverse | TGTGCAGGTTGAAGCCATAG | | circRNA validation |
| Circ-COL1A1-forward | TGGTGACAAGGGTGAGACAG | | circRNA validation |
| Circ-COL1A1-reverse | CTCCTCGCTTTCCTTCCTCT | | circRNA validation |
| Circ-PAPPA2-forward | TGCTGAACGACTTTGACGAC | | circRNA validation |
| Circ-PAPPA2-reverse | ACTGACAGTGTGGGAGCAGTT | | circRNA validation |
| Circ-SLC43A1-forward | TTGGCTACATCATGGACTGG | | circRNA validation |
| Circ-SLC43A1-reverse | AGGAGGCTCCACAGGAAAGT | | circRNA validation |
| Circ-SNRPB-forward | AAGGGAAGAGAAGCGAGTCC | | circRNA validation |
| Circ-SNRPB-reverse | GAAGGTGCCAATGAAGATCC | | circRNA validation |
| Circ-ITGA7-forward | CATGAACAATTTGGGTTCTGC | | circRNA validation |
| Circ-ITGA7-reverse | AGCAGCGACCAATCATATCC | | circRNA validation |
| Circ-PHF21A-forward | CAAATGAAGCAGGATCCACA | | circRNA validation |
| Circ-PHF21A-reverse | TCTCACTGCAGCAATTTTGG | | circRNA validation |
| Circ-GPHN-forward | CGCATGTCTCCTTTTCCTCT | | circRNA validation |
| Circ-GPHN-reverse | CACCTGAACACCACGAGAAA | | circRNA validation |
| Circ-DHRS3-forward | ATGTTCCAGGGCATGAGAGT | | circRNA validation |
| Circ-DHRS3-reverse | TGATGTGTTGGGACTTGAGG | | circRNA validation |
| Circ-BNC2-forward | TGAAAGAGATGCACGTCTGC | | circRNA validation |
| Circ-BNC2-reverse | TTCTCCAAACCGCAGAAACT | | circRNA validation |
| circANKS1B-forward | GAAACCGTCACTGGAGAATTATCA | | circRNA validation |
| circANKS1B-reverse | AAAGCTGCTTCATGAAGTGCAC | | circRNA validation |
| divergent-GAPDH-forward | GTGCTCAACCAGTTGGCACC | | circRNA validation |
| divergent-GAPDH-reverse | AGCCTCGCTCCACCTGACTT | | circRNA validation |
| linear-ANKS1B-forward | CAACCTTCGGAAAAGCAGATG | | linear isoform |
| linear-ANKS1B-reverse | TGGTGCCCGTAAGTGCAGAC | | linear isoform |
| linear-GAPDH-forward | GAACGGGAAGCTCACTGG | | linear isoform |
| linear-GAPDH-reverse | GCCTGCTTCACCACCTTCT | | linear isoform |
| pre-ANKS1B-forward | GCCGATGGATTTTGCTTTTA | | linear isoform |
| pre-ANKS1B-reverse | TTGGAATGCTTACAGGTTGAAA | | linear isoform |
| linear-ANKS1B exon2-3-forward | TTACACCACGCAGCCTTAAA | | linear isoform |
| linear-ANKS1B exon2-3-reverse | TCATTGACCCTGGAATGTGA | | linear isoform |
| linear-ANKS1B exon13-14-forward | TCTCATTGCCTATCCTTCCAA | | linear isoform |
| linear-ANKS1B exon13-14-reverse | TTTCTTCCCATTCCGATGTG | | linear isoform |
| TGF-β1 promoter-forward | GGCTATGGATTTTGCCATGT | | ChIP-qPCR |
| TGF-β1 promoter-reverse | GCCCCACTGTAGATGGTGTC | | ChIP-qPCR |
| GAPDH promoter-forward | AGTGCCTAGGCTCCAGATCA | | ChIP-qPCR |
| GAPDH promoter-reverse | CTCTTCCCACAAATGCTGGT | | ChIP-qPCR |
| TGF-β1-forward | GCAGGGATAACACACTGCAA | | qRT-PCR |
| TGF-β1-reverse | TGAAGCAATAGTTGGTGTCCAG | | qRT-PCR |
| ARF4-forward | GCTGGCAAGACAACCATTCT | | qRT-PCR |
| ARF4-reverse | TTGACCACCAACATCCCATA | | qRT-PCR |
| CSF1-forward | ACCCCAGTTGTCAAGGACAG | | qRT-PCR |
| CSF1-reverse | TTCTGGGACCCAATTAGTGC | | qRT-PCR |
| DOCK3-forward | TTGCAGATCTATGCAGTGACG | | qRT-PCR |
| DOCK3-reverse | AGGCCTGTCATACCGGAACT | | qRT-PCR |
| ESRRG-forward | GACTTGACTCGCCACCTCTC | | qRT-PCR |
| ESRRG-reverse | GTGGTACCCAGAAGCGATGT | | qRT-PCR |
| FGF7-forward | GACATGGATCCTGCCAACTT | | qRT-PCR |
| FGF7-reverse | GGGCTGGAACAGTTCACATT | | qRT-PCR |
| USF1-forward | GTGATCCAGGGTGCTTTCAC | | qRT-PCR |
| USF1-reverse | CCTCTGAGCCCTGGGTAGTA | | qRT-PCR |
| EFNB2-forward | TTATTTGCCCCAAAGTGGAC | | qRT-PCR |
| EFNB2-reverse | TCTGGTTTGGCACAGTTGAG | | qRT-PCR |
| FZD6-forward | ACTCTTGCCACTGTGCCTTT | | qRT-PCR |
| FZD6-reverse | CACAAGATACAAGCCGCTGA | | qRT-PCR |
| LAMA4-forward | GTGCTCCCGGTTACTATGGA | | qRT-PCR |
| LAMA4-reverse | CGCAACGTTCACACTTGAAT | | qRT-PCR |
| ITGA5-forward | GCTGGATGACTTGCTGGTG | | qRT-PCR |
| ITGA5-reverse | ATCATGGCCAGTGAGGGTAA | | qRT-PCR |
| MMP13-forward | ATGACTGAGAGGCTCCGAGA | | qRT-PCR |
| MMP13-reverse | ATTCACCCACATCAGGAACC | | qRT-PCR |
| NFAT5-forward | GTGCTTGCAGCTCCTTTACC | | qRT-PCR |
| NFAT5-reverse | CCTCTTCGGTGTTGATGGAT | | qRT-PCR |
| MTA2-forward | TTTCACGCCATGGATACCTT | | qRT-PCR |
| MTA2-reverse | AGGGCCTCCTCAAATAGCAT | | qRT-PCR |
| SOX5-forward | TCGCTGGAAAGCTATGACAA | | qRT-PCR |
| SOX5-reverse | TGCCTTGTATTCACCAATGC | | qRT-PCR |
| ESRP1-a-forward | TGATTTTGCCCAACACTAGG | | RIP |
| ESRP1-a-reverse | AGATCGCACCACTGCACTC | | RIP |
| ESRP1-b-forward | TCCCAAAGTGCTGGGATTAC | | RIP |
| ESRP1-b-reverse | AAATATCATCCTGGCCCTCA | | RIP |
| ESRP1-c-forward | TGTCTTTGCCAGTTCAGTGTG | | RIP |
| ESRP1-c-reverse | ACTGCGAGTAAAACCCCAAA | | RIP |
| ESRP1-d-forward | TTAGGAATGAGAGTTTACAAAGA | | RIP |
| ESRP1-d-reverse | GTTTTTCTTGCTTTTGTGTTCA | | RIP |
| linear-SYT8-forward | CGACGATTTGTGCCTGTTGG | | RT-PCR |
| linear-SYT8-reverse | GGTGCTGACATCATGCCTCA | | RT-PCR |
| circSYT8-forward | GTGCCTGCAGCTCTCCC | | RT-PCR |
| circSYT8-reverse | CTCCAGGCCATCCACATCAG | | RT-PCR |
| linear-Snail-forward | ATGTATTGAGAATCGGCCCC | | RT-PCR |
| linear-Snail-reverse | AAAAGCAGAGTCAGGCGTTA | | RT-PCR |
| circSnail-forward | CGGACCCACACTGAGTTTACC | | RT-PCR |
| circSnail-reverse | TCCCAGATGAGCATTGGCAG | | RT-PCR |
| ESRP1 promoter 1-forward | TCACCTCTGGCCTCCTTTC | | RT-PCR |
| ESRP1 promoter 1-reverse | AGAGGTAAAGGGCTCGGAGT | | RT-PCR |
| ESRP1 promoter 2-forward | GTGGTTTGAAGGAGCCAATG | | RT-PCR |
| ESRP1 promoter 2-reverse | AATGGCTGAAAGCTTTTCTGTT | | RT-PCR |
| ESRP1 promoter 3-forward | CCGGGCAACTGAATACAAAA | | RT-PCR |
| ESRP1 promoter 3-reverse | GCTAGTGCAAAAGGCGGTAA | | RT-PCR |
| si-circANKS1B#1 | GAAGCCAGAGTGTAACAGA | | siRNA target site |
| si-circANKS1B#2 | GCCAGAGTGTAACAGAAAA | | siRNA target site |
| si-circ/linear ANKS1B | GAAGATGCAACACAAGAAA | | siRNA target site |
| si-linear-ANKS1B | CCAGTCGTGTGATTACAAA | | siRNA target site |
| si-USF1#1 | CCAGTGATGATGCAGTTGA | | siRNA target site |
| si-USF1#2 | GCTTGTCTGAAGAACTGCA | | siRNA target site |
| si-TGF-β1#1 | GCAGAGTACACACAGCATA | | siRNA target site |
| si-TGF-β1#2 | CCACCATTCATGGCATGAA | | siRNA target site |
| si-ESRP1#1 | GGACAGCATTGCCCTATTA | | siRNA target site |
| si-ESRP1#2 | CAGTGAGCAATGAACTGAA | | siRNA target site |
| si-ESRP2#1 | GGGTAAGCGATACATTGAA | | siRNA target site |
| si-ESRP2#2 | GGGAAGTCAAGACAATGGT | | siRNA target site |
| si-ATXN1#1 | CAGATCCATTTACACTTTA | | siRNA target site |
| si-ATXN1#2 | GAAGTCTCCATGGTGAAGT | | siRNA target site |
| si-QKI#1 | GGGACCTATTGTTCAGTTA | | siRNA target site |
| si-QKI#2 | GCTGCTCCAAGGATCATTA | | siRNA target site |
| si-WT1#1 | CAGGCTGCAATAAGAGATA | | siRNA target site |
| si-WT1#2 | CAACCACTCATTCAAGCAT | | siRNA target site |
| si-BICC1#1 | GGAAGAAACTTGAGGCCAT | | siRNA target site |
| si-BICC1#2 | GCCCAAGCCACATTAACTA | | siRNA target site |
| si-APOBEC3B#1 | CTCAGTACCACGCAGAAAT | | siRNA target site |
| si-APOBEC3B#2 | CTGCCCGCATCTATGATTA | | siRNA target site |
| si-IFIT#1 | GCTGAGCTTAAGATAAACA | | siRNA target site |
| si-IFIT#2 | GAAGCCCTGAAGAGCTTAA | | siRNA target site |
| si-NANOS3#1 | CCTGTGCTCTTTCTGCAAA | | siRNA target site |
| si-NANOS3#2 | GCCCTCAATAAATGCTTAT | | siRNA target site |
| si-IGF2BP1#1 | GTGCCATTATTGGCAAGGA | | siRNA target site |
| si-IGF2BP1#2 | GGAGGCCTATGAGAATGAT | | siRNA target site |
| si-NOVA1#1 | GGTTCTCATACCTAGTTAT | | siRNA target site |
| si-NOVA1#2 | GGTGCAAGGATACAGATCT | | siRNA target site |
| si-MSI1#1 | GAACGAAGAAGATCTTTGT | | siRNA target site |
| si-MSI1#2 | GCCATGCTGATGTTTGACA | | siRNA target site |
| si-MEX3B#1 | GGCTCCTTAAAGAAACGCT | | siRNA target site |
| si-MEX3B#2 | GCGAAGACCAATACTTACA | | siRNA target site |
| si-NOVA2#1 | CCAAGTCCAAAGACTTCTA | | siRNA target site |
| si-NOVA2#2 | GCATCCAGATCTCCAAGAA | | siRNA target site |
| circANKS1B probe | TCCCCTTTTCTGTTACACTCTGGCT | | FISH |
| miR-148a-3p probe | ACAAAGTTCTGTAGTGCACTGA | | FISH |
| miR-152-3p probe | CCAAGTTCTGTCATGCACTGA | | FISH |
| Biotin-circANKS1B probe | TTCTGTTACACTCTGGCTTC | | RNA pull down |
| Biotin-miR-148a-3p probe  sense | UCAGUGCACUACAGAACUUUGU | | RNA pull down |
| Biotin-miR-148a-3p probe anti-sense | ACAAAGUUCUGUAGUGCACUGA | | RNA pull down |
| Biotin-miR-152-3p probe sense | UCAGUGCAUGACAGAACUUGG | | RNA pull down |
| Biotin-miR-152-3p probe anti-sense | CCAAGUUCUGUCAUGCACUGA | | RNA pull down |
| Biotin-miR-148a-3p mutant probe sense | UCCAGAUGCUACAGAACUUUGU | | RNA pull down |
| Biotin-miR-148a-3p mutant probe anti-sense | ACAAAGUUCUGUAGCAUCUGGA | | RNA pull down |
| Biotin-miR-152-3p mutant probe sense | UCUCGUACUGACAGAACUUGG | | RNA pull down |
| Biotin-miR-152-3p mutant probe anti-sense | CCAAGUUCUGUCAGUACGAGA | | RNA pull down |
|  | | | |
| **miRNA mimics and inhibitors** | | | |
| mimics NC-sense | | UCACAACCUCCUAGAAAGAGUAGA | |
| mimics NC-anti-sense | | UCUACUCUUUCUAGGAGGUUGUGA | |
| miR-148a-3p mimics-sense | | UCAGUGCACUACAGAACUUUGU | |
| miR-148a-3p mimics-anti-sense | | ACAAAGUUCUGUAGUGCACUGA | |
| miR-152-3p mimics-sense | | UCAGUGCAUGACAGAACUUGG | |
| miR-152-3p mimics-anti-sense | | CCAAGUUCUGUCAUGCACUGA | |
| anti NC | | UCACAACCUCCUAGAAAGAGUAGA | |
| anti miR-148a-3p | | ACAAAGUUCUGUAGUGCACUGA | |
| anti miR-152-3p | | CCAAGUUCUGUCAUGCACUGA | |


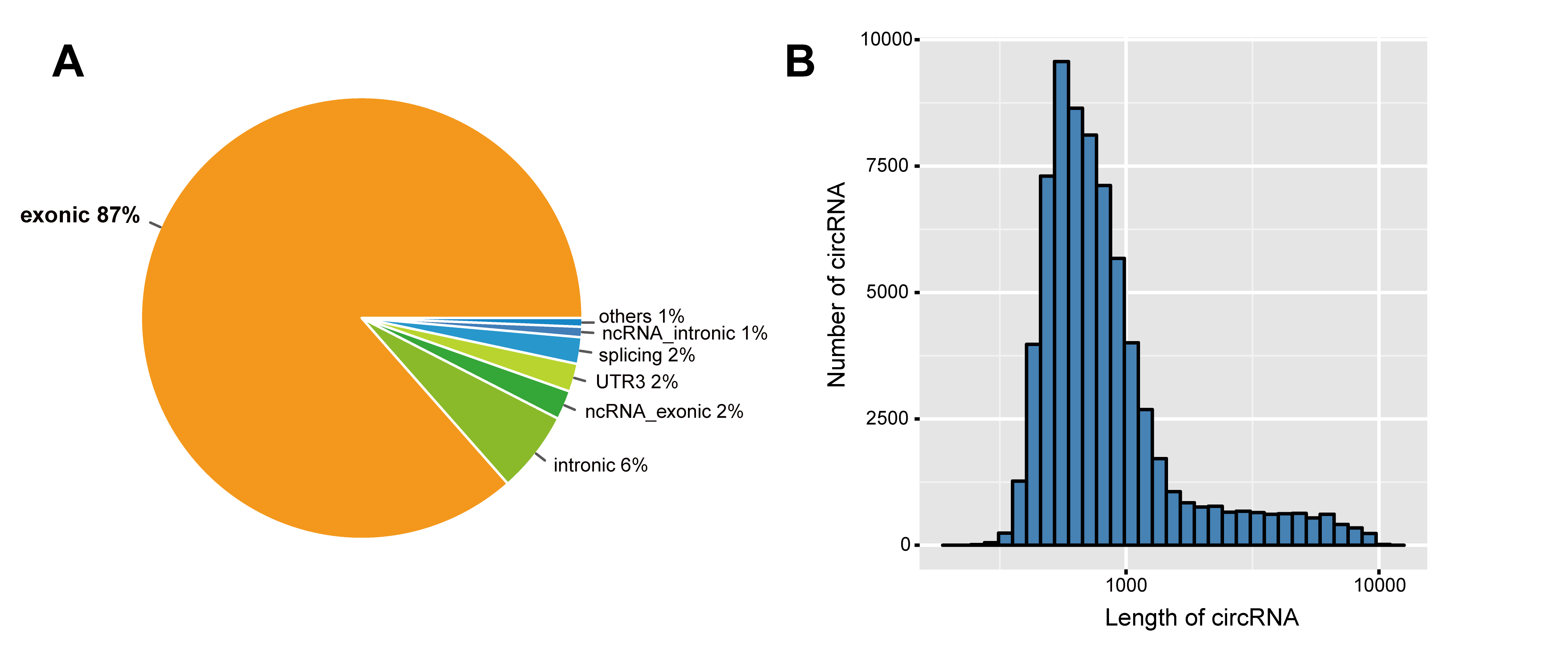


**Figure S1.** (A) Genomic origin of circRNAs (n = 69,815) identified in human breast tissues. 87% were derived from exons, and the others were derived from introns, intergenic region and 3′ or 5′ UTR, etc. (B) The length distribution for circRNAs (n = 69,815) identified in human breast tissues. Most of the circRNAs are less than 1,300 nucleotides (nt) in length.


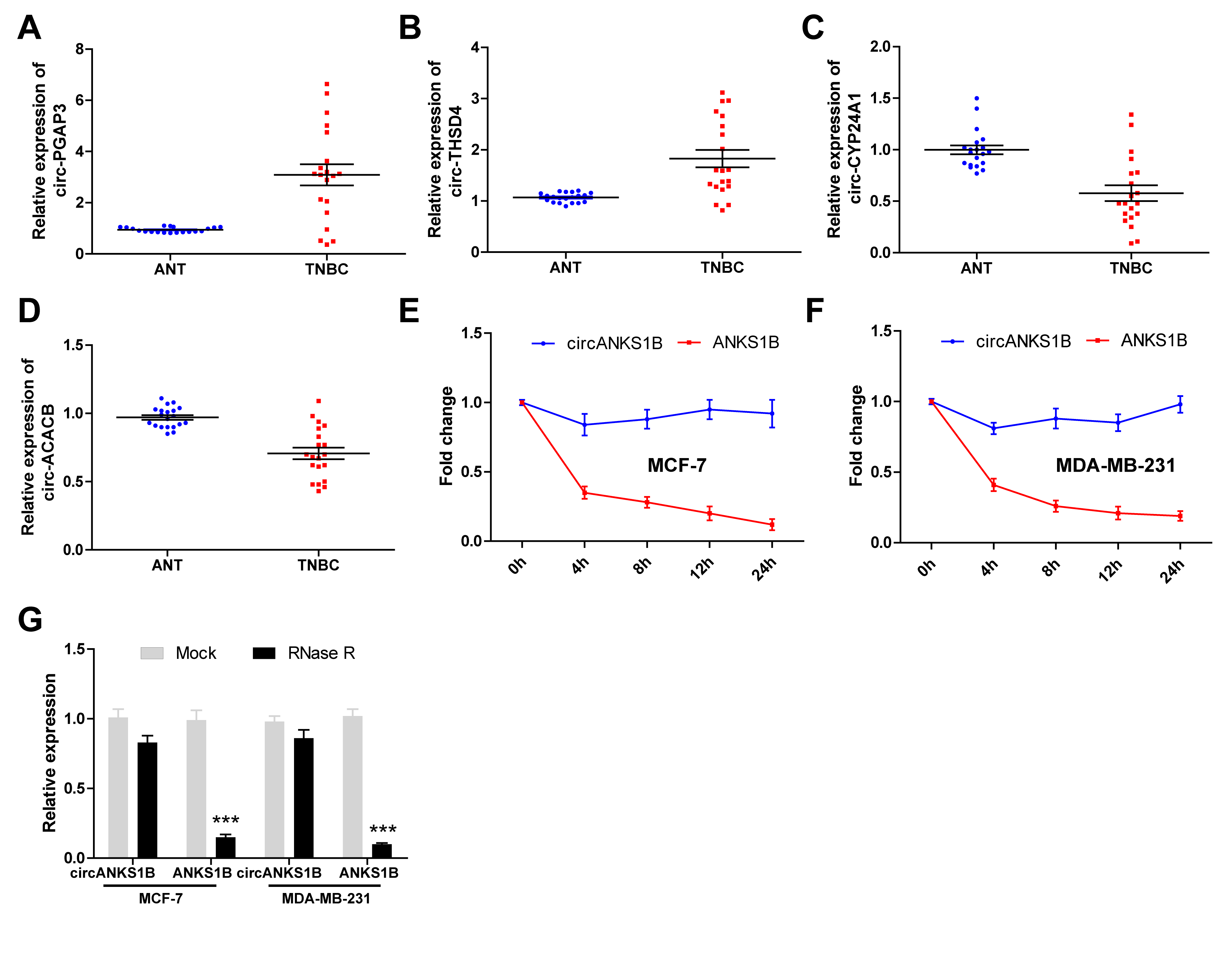


**Figure S2.** (A-D) qRT-PCR analysis of the screened top ten most increased and decreased circRNAs in twenty pairs of TNBC and adjacent normal tissues. Four circRNAs (circ-PGAP3, circ-THSD4, circ-CYP24A1 and circ-ACACB) were validated to be significantly dysregulated. (E-F) qRT–PCR analysis of the abundance of circANKS1B and ANKS1B mRNA in MCF-7 and MDA-MB-231 cells treated with Actinomycin D at the indicated time points. (G) qRT–PCR analysis of circANKS1B and ANKS1B mRNA after treatment with RNase R in MCF-7 and MDA-MB-231 cells. Data were represented as means ± S.D. of at least three independent experiments. ********p* < 0.001.


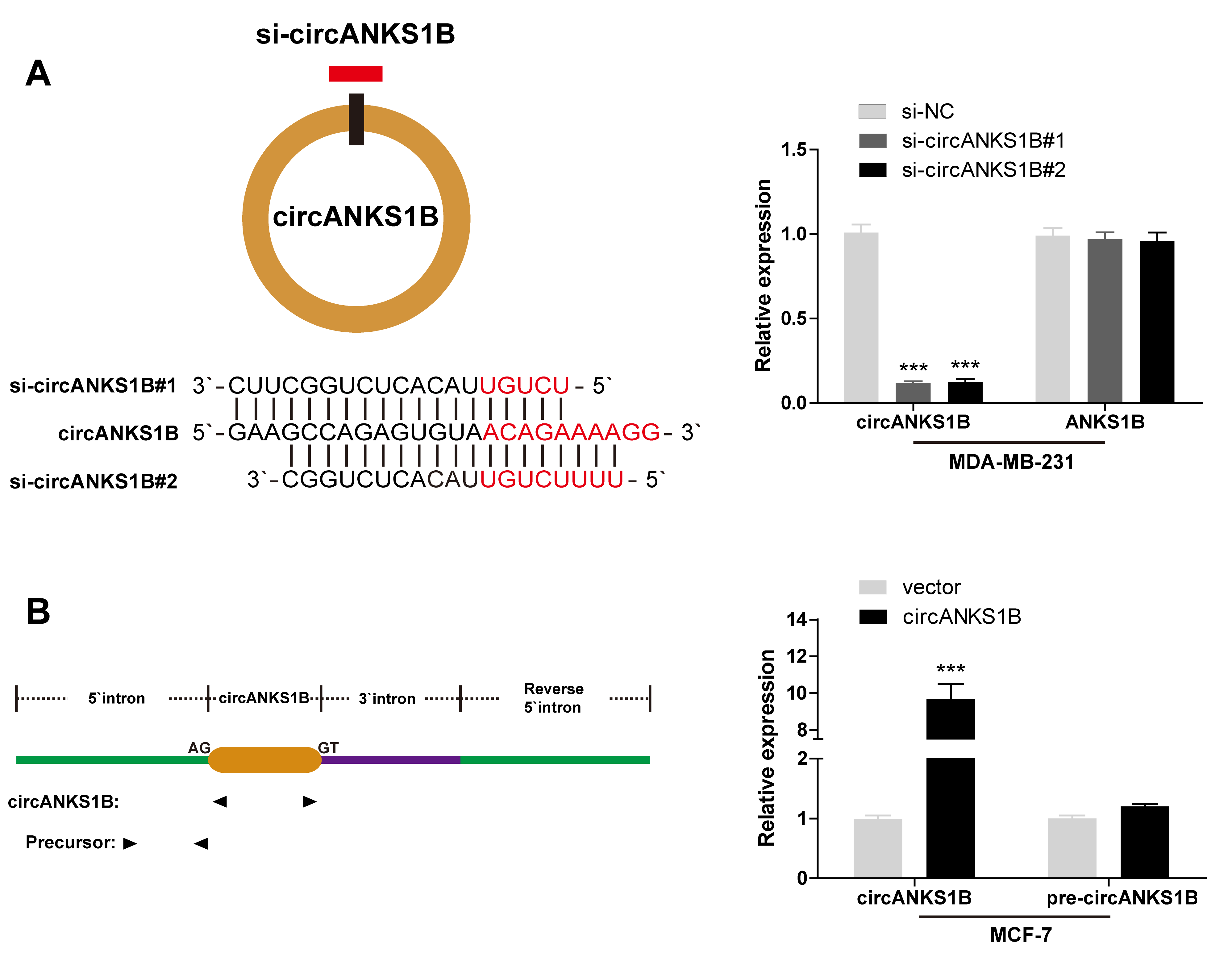


**Figure S3.** (A) Schematic of two siRNA targeting circANKS1B junction site (left). These two siRNA effectively silenced circANKS1B expression in MDA-MB-231 cells, whereas had no effect on linear ANKS1B expression (right). (B) Schematic of construction of circANKS1B overexpression vector, the sequence of the 5′-flanking intron was copied and inversely inserted the downstream of 3′-flanking intron (left). The overexpression vector effectively increased circANKS1B expression in MCF-7 cells, while did not affect its precursor expression. Data were represented as means ± S.D. of at least three independent experiments. ********p* < 0.001.


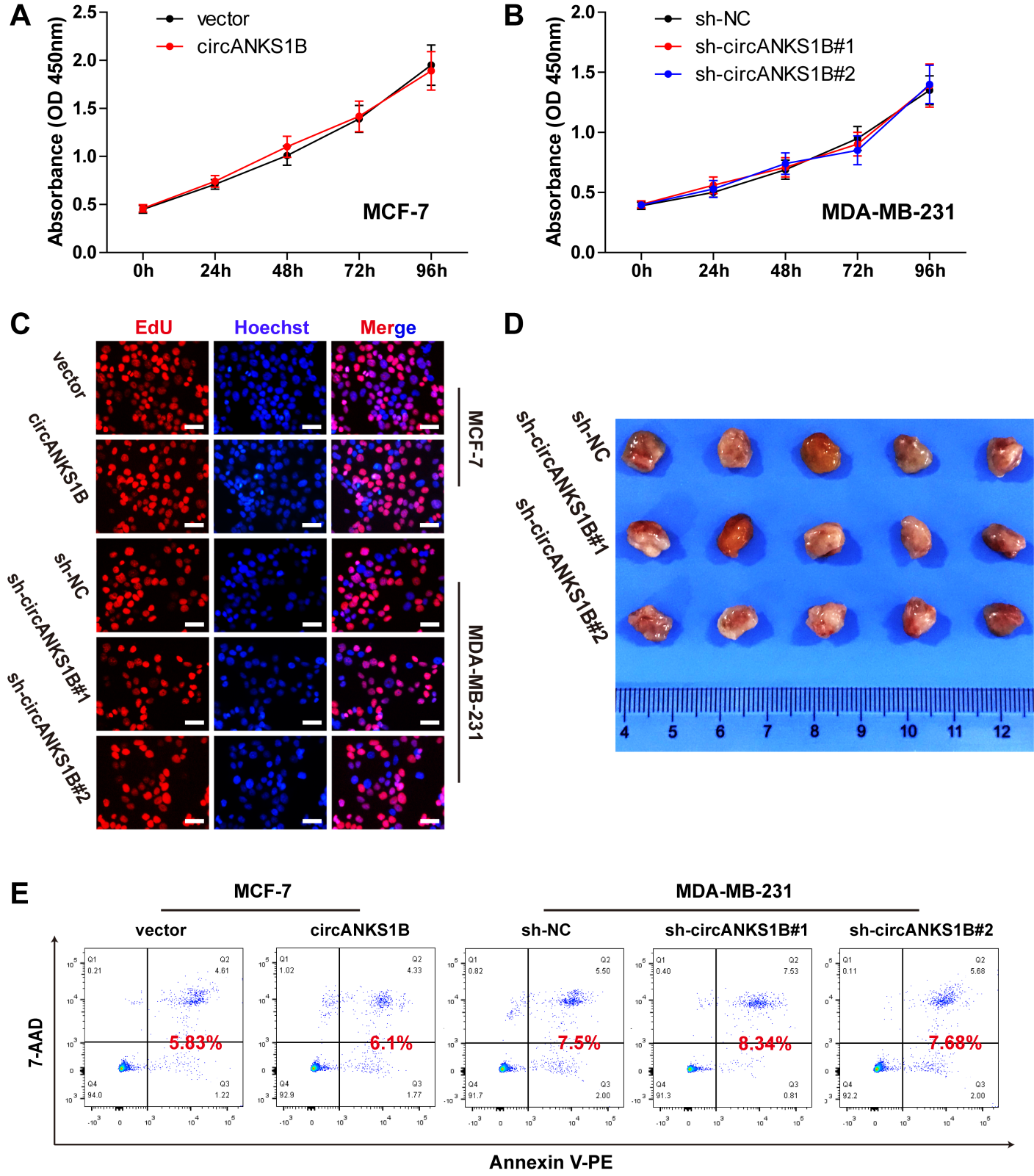


**Figure S4.** (A-C) CCK-8 and EdU analysis of the proliferative abilities of MCF-7 cells with circANKS1B overexpression and MDA-MB-231 cells with circANKS1B knockdown. Scale bar = 20 μm. (D) The images of tumor-bearing nude mice from the indicated treatment groups (n = 5 for each group) on the 49th day. (E) Annexin V-PE/7-AAD double staining analysis of apoptosis of MCF-7 cells with circANKS1B overexpression and MDA-MB-231 cells with circANKS1B knockdown.

Data were represented as means ± S.D. of at least three independent experiments.


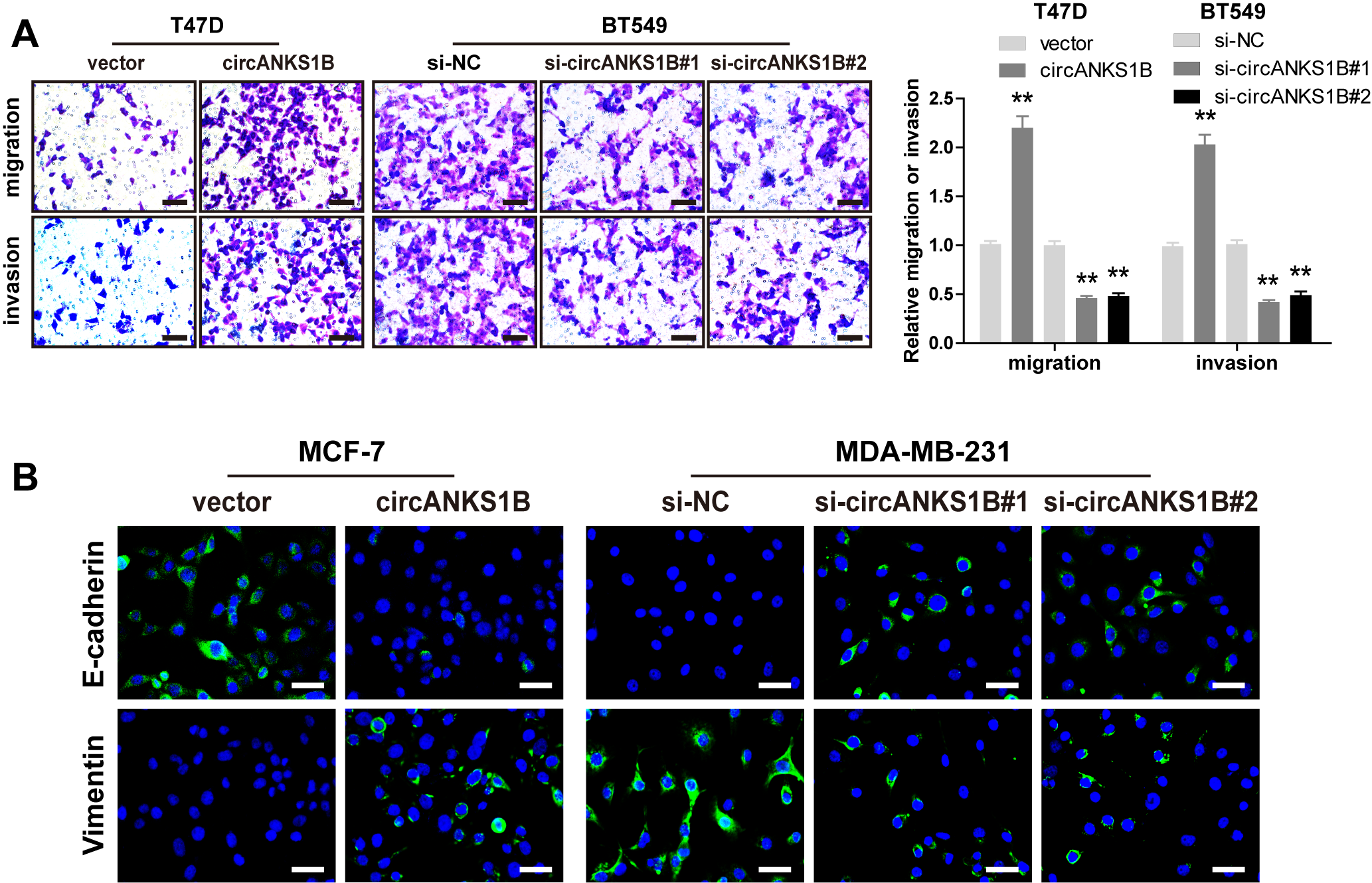


**Figure S5.** (A) Transwell migration and matrigel invasion assays for T47D cells with circANKS1B overexpression and BT549 cells with circANKS1B knockdown. Scale bar = 20 μm. (B) Immunofluorescence analysis of E-cadherin and Vimentin in circANKS1B-overexpressing MCF-7 cells and circANKS1B knockdown MDA-MB-231 cells. Scale bar = 20 μm. Data were represented as means ± S.D. of at least three independent experiments. *******p* < 0.01.


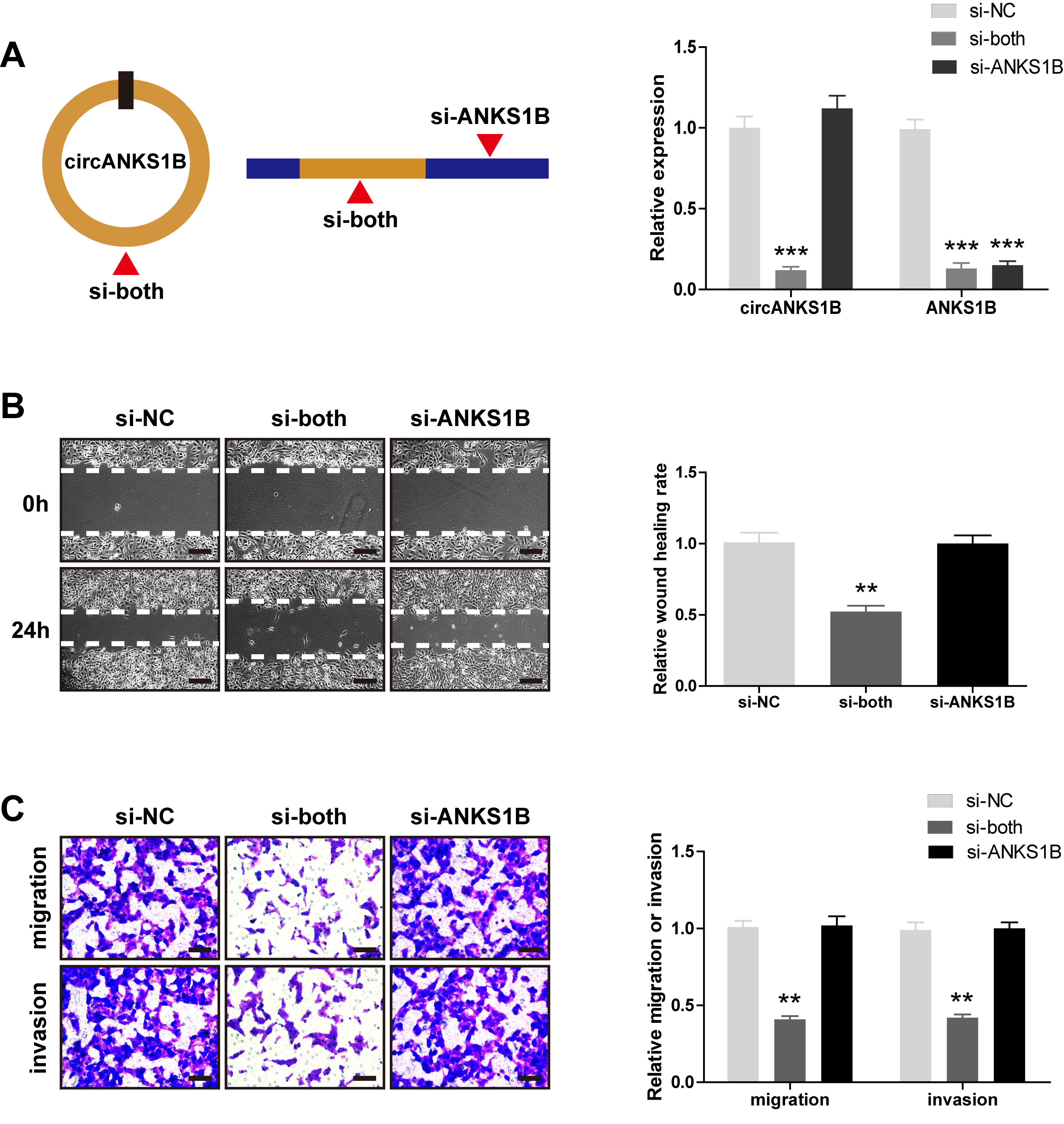


**Figure S6.** (A) Schematic illustration showing two targeted siRNAs. si-ANKS1B targets the ANKS1B linear transcript, si-both targets both the linear ANKS1B and circANKS1B (left). Their respective inhibitory effects were verified by qRT-PCR (right). (B-C) Wound healing, transwell migration and invasion assays for MDA-MB-231 cells transfected with si-NC, si-both or si-ANKS1B. Scale bar = 20 μm. Data were represented as means ± S.D. of at least three independent experiments. *******p* < 0.01, ********p* < 0.001.


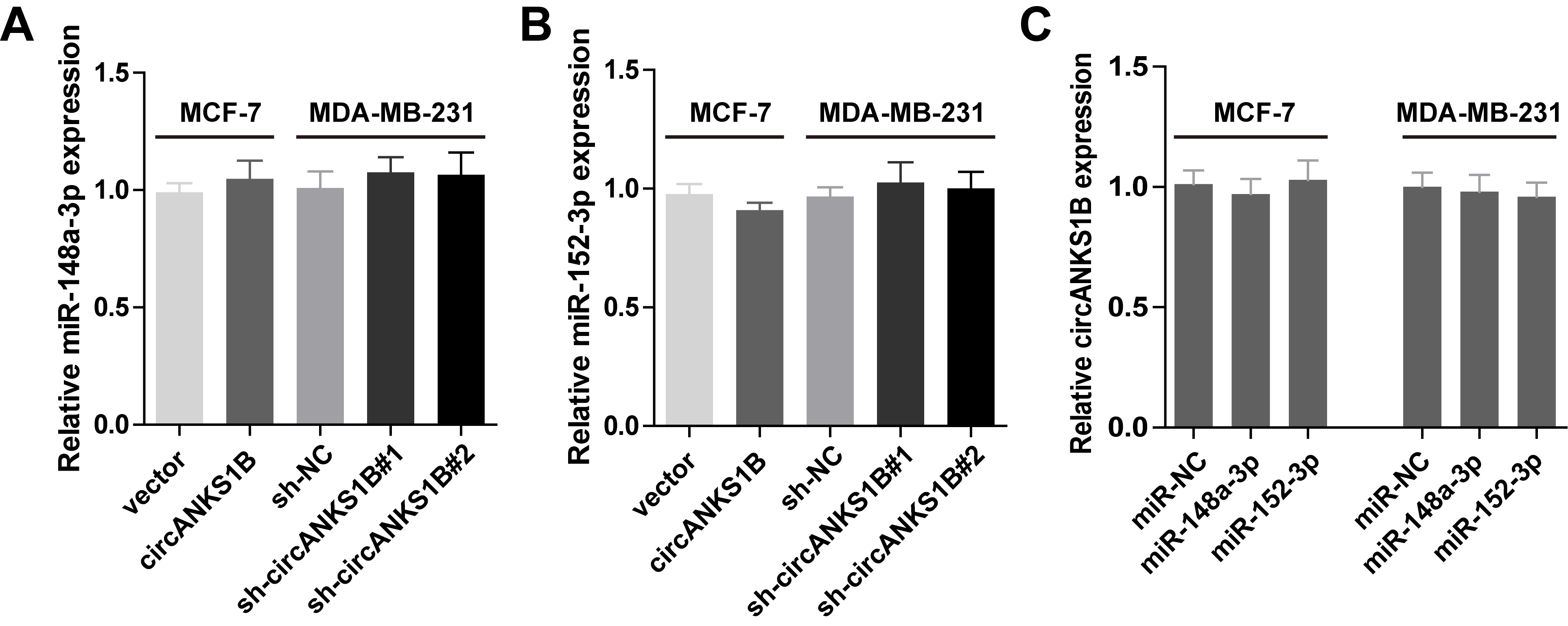


**Figure S7.** (A-B) qRT-PCR analysis of miR-148a-3p and miR-152-3p in circANKS1B-overexpressing MCF-7 cells and circANKS1B knockdown MDA-MB-231 cells. (C) qRT-PCR analysis of circANKS1B in MCF-7 and MDA-MB-231 cells transfected with miR-148a/152-3p mimics or control mimics. Data were represented as means ± S.D. of at least three independent experiments.


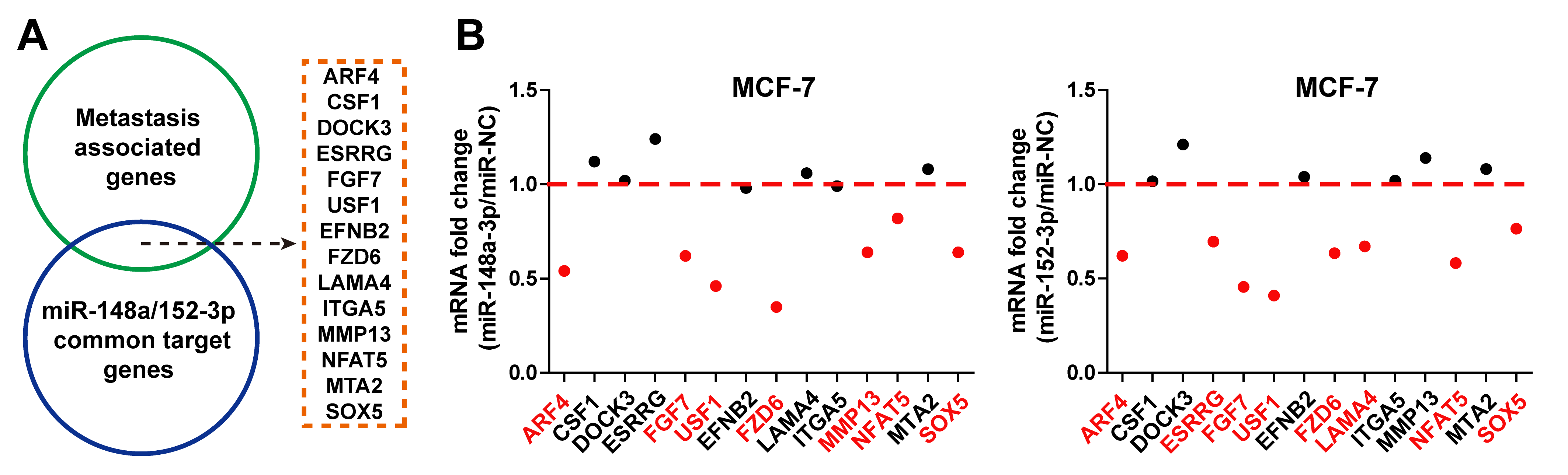


**Figure S8.** (A) Schematic of the selection of these 14 metastasis-related genes targeted by miR-148a-3p and miR-152-3p. (B) qRT-PCR analysis of these 14 metastasis-related genes in MCF-7 with miR-148a-3p or miR-152-3p overexpression. Solid red and black circles indicate the genes regulated and non-regulated by miR-148a-3p or miR-152-3p, respectively.


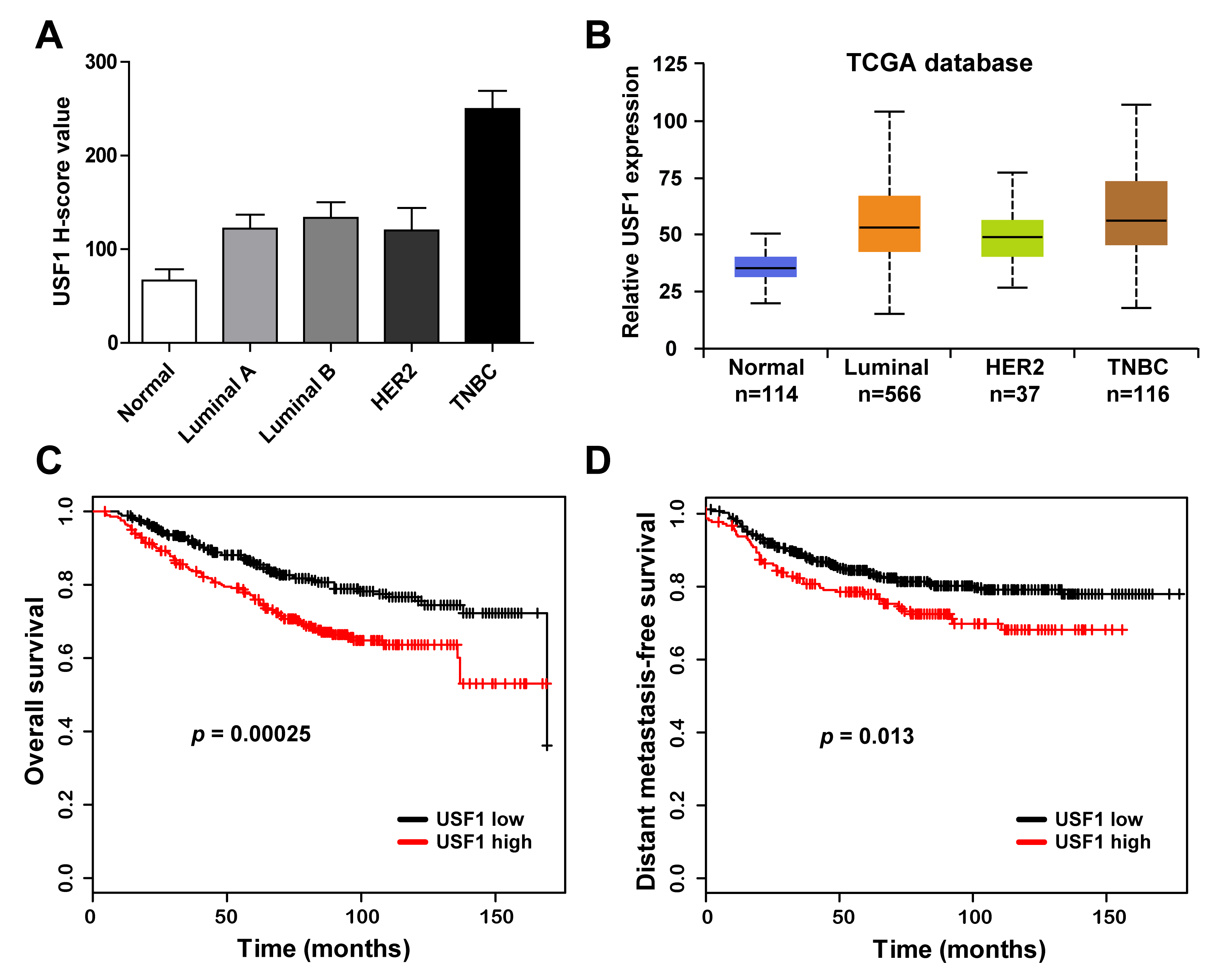


**Figure S9.** (A) IHC analysis of USF1 in normal tissues (n=40) and breast cancer tissues (n=165). (B) The expression of USF1 mRNA in breast cancer tissues from TCGA database. (C) The overall survival curves in breast cancer patients with low and high USF1 expression from KM-plotter database (http://kmplot.com/analysis/). (D) The distant metastasis-free survival curves in breast cancer patients with low and high USF1 expression from KM-plotter database.


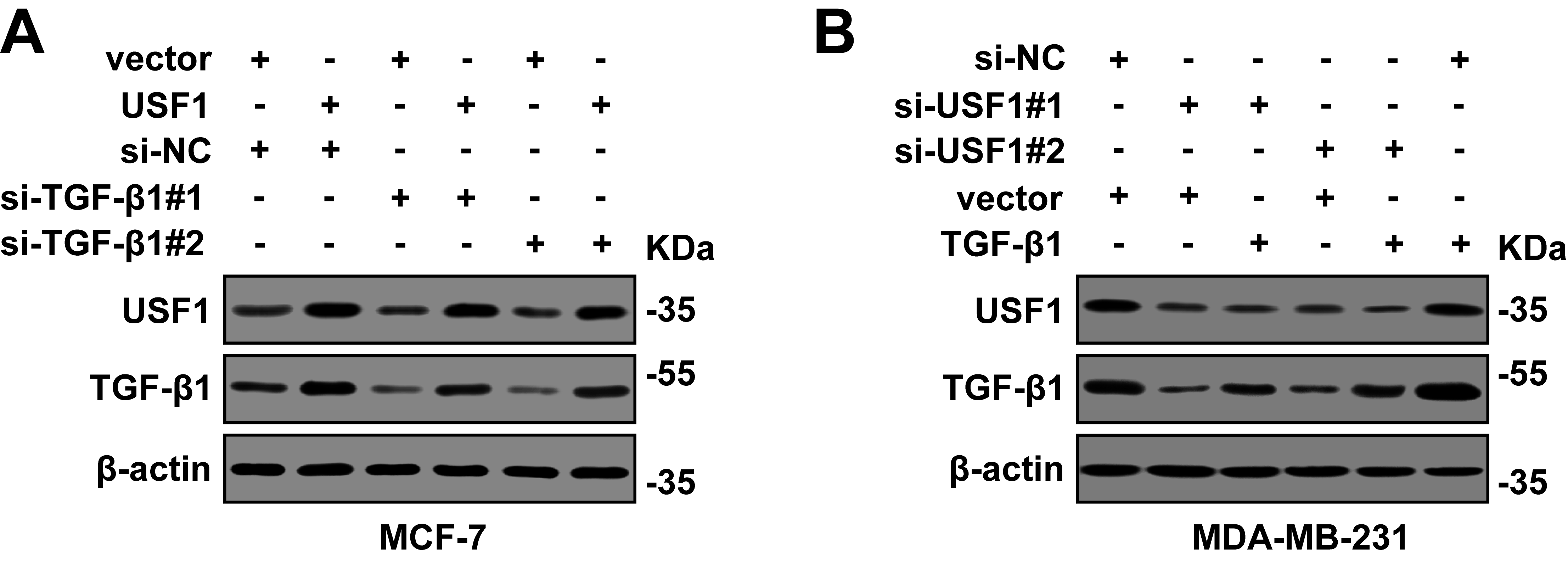


**Figure S10.** Immunoblot analysis of USF1 and TGF-β1 in MCF-7 (A) or MDA-MB-231 cells (B) in the indicated groups. β-actin was used as a loading control.


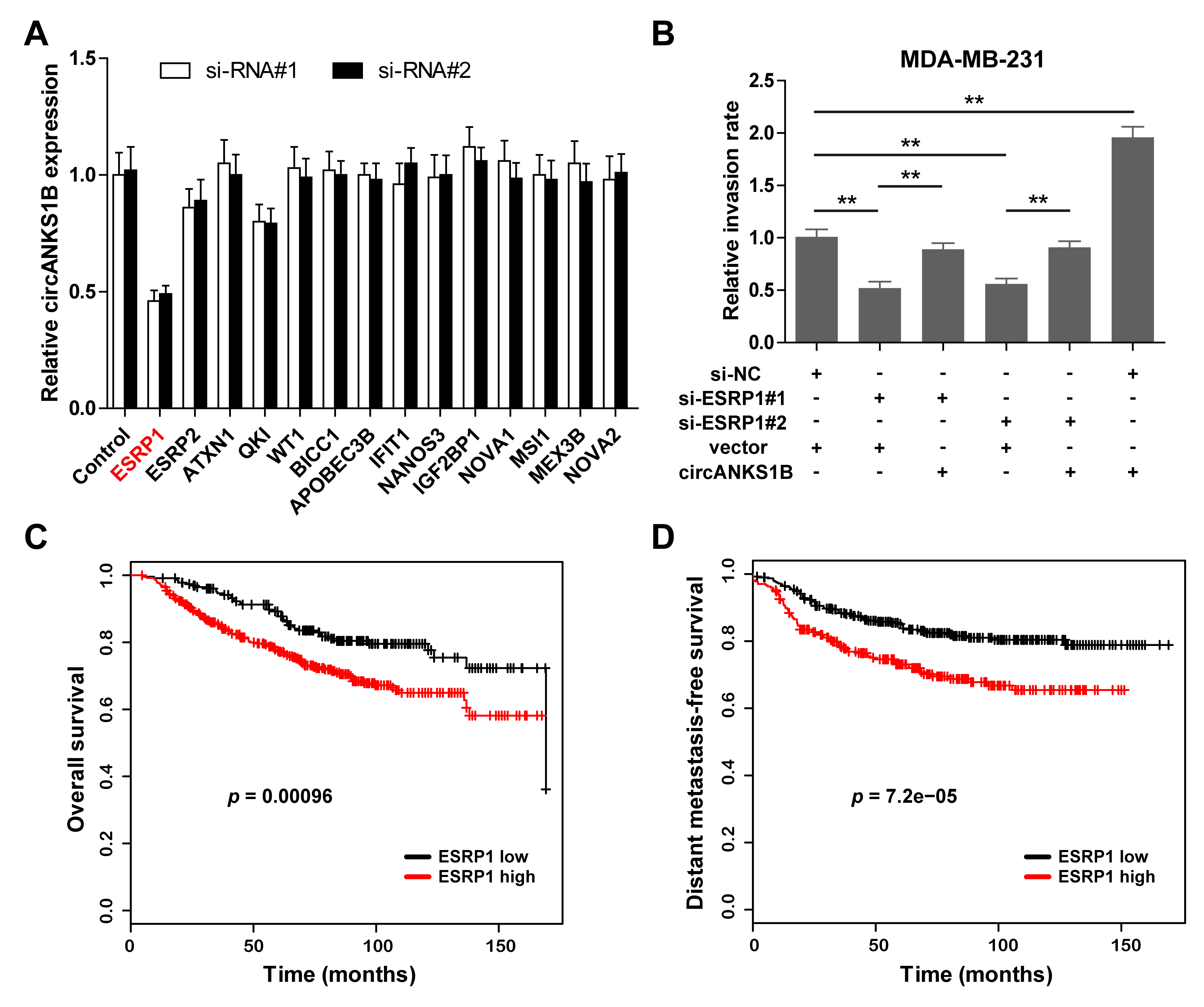


**Figure S11.** (A) qRT-PCR analysis of circANKS1B expression in MDA-MB-231 cells transfected with the indicated siRNAs. (B) Transwell invasion assay for MDA-MB-231 cells co-transfected with si-ESRP1 or si-NC and circANKS1B or control vector. (C-D) The overall and distant metastasis-free survival curves in breast cancer patients with low and high ESRP1 expression from KM-plotter database (http://kmplot.com/analysis/). Data were represented as means ± S.D. of at least three independent experiments.


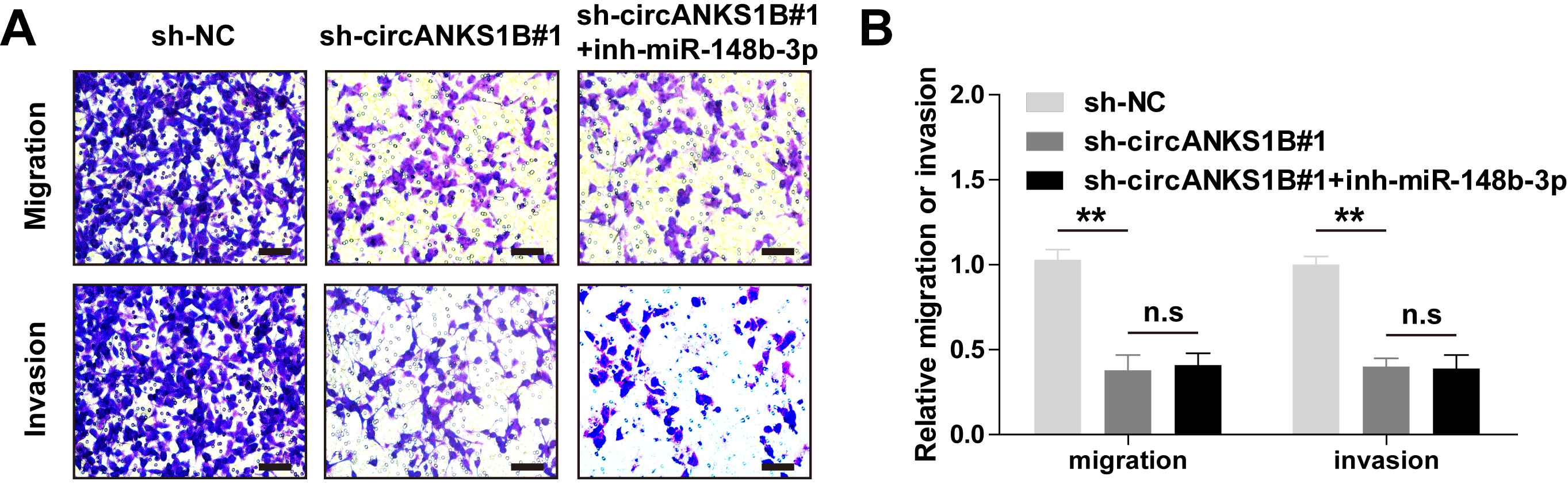


**Figure S12.** (A-B) Transwell migration and invasion assays for circANKS1B silencing MDA-MB-231 cells transfected with miR-148b-3p inhibitors. Data were represented as means ± S.D. of at least three independent experiments. Scale bar = 20 μm. *******p* < 0.01, n.s = not significant.
